# mTORC1 Prevents Preosteoblast Differentiation through the Notch Signaling Pathway

**DOI:** 10.1371/journal.pgen.1005426

**Published:** 2015-08-04

**Authors:** Bin Huang, Yongkui Wang, Wenhao Wang, Juan Chen, Pinglin Lai, Zhongyu Liu, Bo Yan, Song Xu, Zhongmin Zhang, Chun Zeng, Limin Rong, Bin Liu, Daozhang Cai, Dadi Jin, Xiaochun Bai

**Affiliations:** 1 Academy of Orthopedics, Guangdong Province, Department of Orthopedics, The Third Affiliated Hospital, Southern Medical University, Guangzhou, China; 2 Department of Cell Biology, School of Basic Medical Science, Southern Medical University, Guangzhou, China; 3 Department of Spine Surgery, The Third Affiliated Hospital, Sun Yat-sen University, Guangzhou, China; Stanford University School of Medicine, UNITED STATES

## Abstract

The mechanistic target of rapamycin (mTOR) integrates both intracellular and extracellular signals to regulate cell growth and metabolism. However, the role of mTOR signaling in osteoblast differentiation and bone formation is undefined, and the underlying mechanisms have not been elucidated. Here, we report that activation of mTOR complex 1 (mTORC1) is required for preosteoblast proliferation; however, inactivation of mTORC1 is essential for their differentiation and maturation. Inhibition of mTORC1 prevented preosteoblast proliferation, but enhanced their differentiation *in vitro* and in mice. Activation of mTORC1 by deletion of *tuberous sclerosis 1* (*Tsc1*) in preosteoblasts produced immature woven bone in mice due to excess proliferation but impaired differentiation and maturation of the cells. The mTORC1-specific inhibitor, rapamycin, restored these *in vitro* and *in vivo* phenotypic changes. Mechanistically, mTORC1 prevented osteoblast maturation through activation of the STAT3/p63/Jagged/Notch pathway and downregulation of Runx2. Preosteoblasts with hyperactive mTORC1 reacquired the capacity to fully differentiate and maturate when subjected to inhibition of the Notch pathway. Together, these findings identified the role of mTORC1 in osteoblast formation and established that mTORC1 prevents preosteoblast differentiation and maturation through activation of the Notch pathway.

## Author Summary

The coordinated activities of osteoblasts and osteoclasts in bone deposition and resorption form the internal structure of bone. Disruption of the balance between bone formation and resorption results in loss of bone mass and causes bone diseases such as osteoporosis. Current therapies for osteoporosis are limited to anti-resorptive agents, while bone diseases due to reduced osteoblast activity, such as senile osteoporosis, urgently require targeted treatment and novel strategies to promote bone formation. mTORC1 has emerged as a critical regulator of bone formation and is therefore a potential target in the development of novel bone-promoting therapeutics. Identifying the detailed function of mTORC1 in bone formation and clarifying the underlying mechanisms may uncover useful therapeutic targets. In this study, we reveal the role of mTORC1 in osteoblast formation. mTORC1 stimulated preosteoblast proliferation but prevented their differentiation and attenuated bone formation via activation of the Notch pathway. Pharmaceutical coordination of the pathways and agents in preosteoblasts may be beneficial in bone formation.

## Introduction

The skeleton is a highly specialized and dynamic structure undergoing constant remodeling [1]. The remodeling process is executed by temporary cellular structures that comprise teams of coupled osteoblasts and osteoclasts. The rate of genesis as well as death of these two cell types is vital for the maintenance of bone homeostasis [2], and common metabolic bone disorders such as osteoporosis are largely caused by a derangement in the proliferation, differentiation or apoptosis of these cells [3].

Osteoblasts, which are the chief bone-making cells, differentiate and produce bone matrix during skeletal development [4]. The differentiation process of osteoblasts is often divided into stages of mesenchymal progenitors, preosteoblasts and osteoblasts (often called mature osteoblasts) [5]. Osteoblasts are often characterized by the expression of osteocalcin, while preosteoblasts are usually considered to express the transcription factor Runx2 or both Runx2 and osterix (Osx). Preosteoblasts have been shown to actively divide *in vitro* and are multipotent in differentiating, thus proliferative expansion and osteoblastic differentiation of preosteoblasts are essential for bone formation. Understanding the intracellular signaling pathways that control preosteoblast proliferation and differentiation is critical for preventing bone loss-related disease caused by impaired bone formation such as senile osteoporosis.

The mechanistic target of rapamycin (mTOR) is a conserved Ser/Thr kinase nucleating at least two distinct multi-protein complexes, mTOR complex 1 (mTORC1) and mTOR complex 2 (mTORC2) [6]. mTORC1 uniquely contains raptor and is the sensitive target of rapamycin, it integrates both intracellular and extracellular signals, including growth factors, nutrients, energy levels, and cellular stress [7]. The tuberous sclerosis 1 (TSC1)-TSC2-TBC1D7 (TSC-TBC) complex is the major upstream inhibitory regulator of mTORC1 [8], and loss of this complex causes cells and tissues to display constitutive mTORC1 activation. TSC-TBC accelerates the intrinsic rate of GTP hydrolysis of Rheb, converting Rheb from the GTP-bound (active) to the GDP-bound (inactive) form. The active GTP-bound form of Rheb directly interacts with mTORC1 to stimulate its activity. mTORC1 phosphorylates the translational regulators, eukaryotic initiation factor 4E-binding protein-1 (4E-BP1) and S6 kinase 1 (S6K1), to regulate biosynthesis of proteins and modulates autophagy, biosynthesis of lipids and organelles and mitochondrial metabolism as well, through which mTORC1 exerts an essential role in regulating cell metabolism, survival, growth and proliferation [6,9].

mTORC1 signaling has emerged as a critical regulator of bone formation. Patients with tuberous sclerosis due to mutation in *TSC1/2* present with sclerotic and lytic bone changes [10,11,12,13,14]. Moreover, mTOR has recently been identified among genes and pathways that are connected with human skeletal growth [15]. However, results from *in vitro* and *in vivo* studies on the function of mTORC1 in osteoblast lineage are inconsistent. The mTORC1 inhibitor, rapamycin, showed controversial capacity to influence the differentiation of various osteoblastic lineage cell lines *in vitro* [16,17,18,19,20,21,22] and demonstrated inconsistent potential for bone formation *in vivo* [23,24,25]. In addition to an undefined function in the osteoblast lineage, the mechanisms through which mTORC1 modulates osteoblast differentiation and bone formation are unknown. Using conditional *Tsc1* knockout cell and mouse models, we demonstrated that mTORC1 activation is crucial for preosteoblast proliferation, but prevents their differentiation and maturation.

Notch signaling mediates communication between neighboring cells to control cell fate decision [26, 27]. Notch has been reported to be positively regulated by mTORC1 in various cell lines and be responsible for the impaired cell differentiation by mTORC1 [28, 29, 30]. We determined here that it is true in osteoblast lineage cells as well. Our mechanistic studies revealed that Notch signaling is strongly activated by mTORC1 to inhibit osteoblastic transcription factor Runx2 and prevent preosteoblast maturation and bone formation.

## Results

### mTORC1 is activated during preosteoblast proliferation but is suppressed during their differentiation

**Fig 1:**
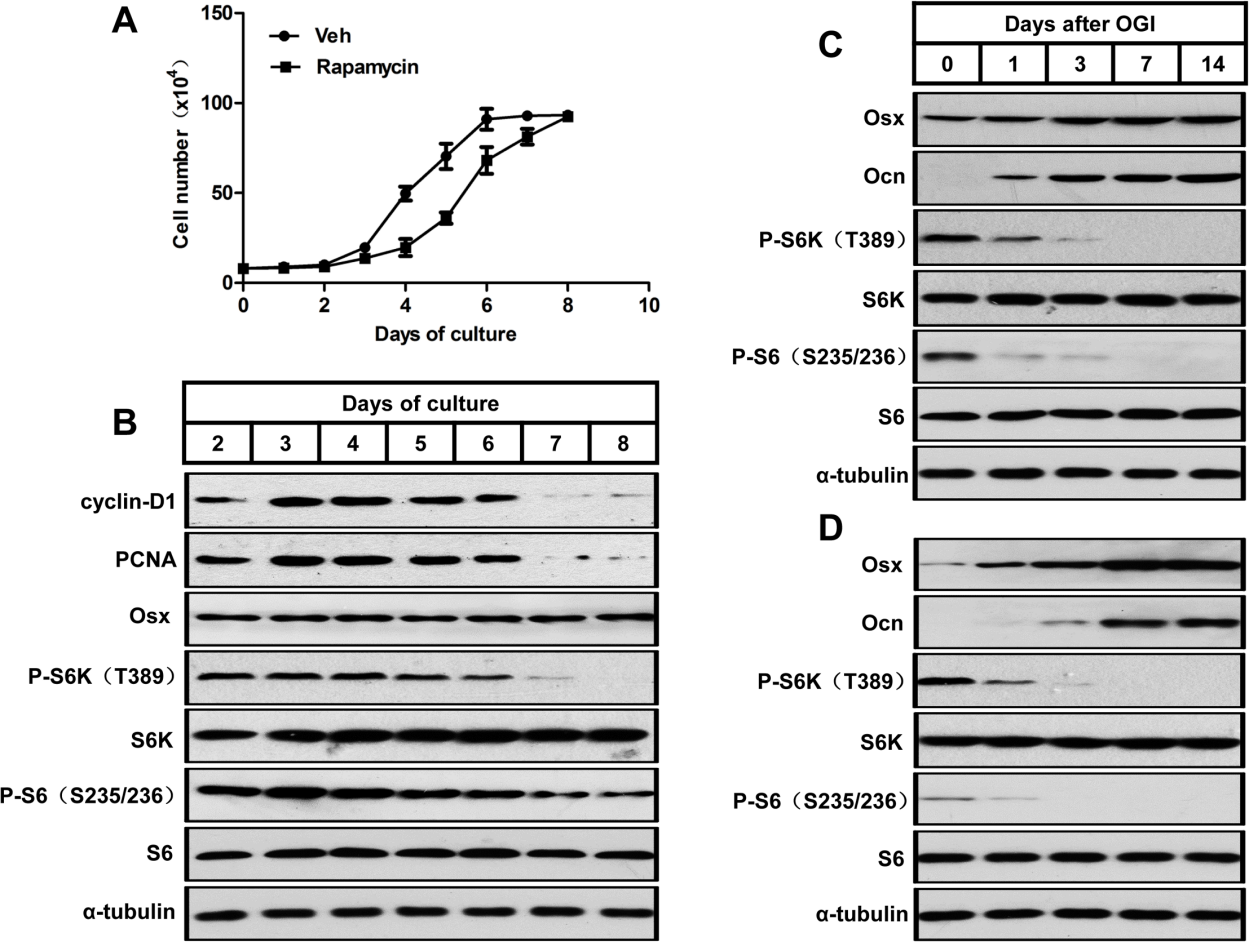
mTORC1 is activated during preosteoblast proliferation but is suppressed during their differentiation. (A) Growth curve of proliferating MC3T3-E1 cells treated with vehicle (Veh) or rapamycin (0.1nM). (B) Western blot analysis of proliferative markers (cyclin D1 and PCNA), Osx and mTORC1 activity (indicated by P-S6K (T389) and P-S6 (S235/236)) during MC3T3-E1 proliferation. (C) Western blot analysis of osteoblastic markers (Osx and Ocn) and mTORC1 activity during differentiation of MC3T3-E1 cells. (D) Western blot analysis of osteoblastic markers (Osx and Ocn) and mTORC1 activity during differentiation of FRC. Osx: osterix; Ocn: osteocalcin; FRC: fetal rat calvarial cells, OGI: osteogenic induction.

mTORC1 is known to promote cell proliferation in many types of cells. To examine the relationship between preosteoblast proliferation and mTORC1 activity, we analyzed the level of mTORC1 during the proliferative expansion of MC3T3-E1 cells, a murine preosteoblast cell line [31], and subsequent cessation of growth. We counted cells each day to monitor their growth and observed that the cells reached confluence after 6–7 days, when proliferation ceased due to contact inhibition (Fig 1A). Western blot analysis reflected this growth inhibition as a decrease in the levels of the cell cycle markers, cyclin D1 and proliferative cell nuclear antigen (PCNA). As expected, the high level of mTORC1 activity (indicated by P-S6K (T389) and P-S6 (S235/236)) decreased when proliferation of MC3T3-E1 cells slowed down and eventually ceased (Fig 1B). Cells did not differentiate during this growth period, as the level of osterix (Osx, a marker of early osteoblasts) was unchanged throughout. These data indicate that the level of mTORC1 activity was positively correlated with the rate of preosteoblast proliferation.

We then induced osteoblastic differentiation in confluent MC3T3-E1 cells. The induced cells showed a decreased mTORC1 activity during osteoblast differentiation in parallel with increases in markers of osteoblast differentiation (i.e. osterix (Osx) and osteocalcin (Ocn)) (Fig 1C). A similar pattern of mTORC1 activity was observed in osteoblastic induced fetal rat calvarial cells (Fig 1D), which implicated that mTORC1 activity is not required for osteoblastic differentiation of preosteoblasts.

### Inactivation of mTORC1 prevents preosteoblast proliferation but enhances their differentiation *in vitro* and *in vivo*

**Fig 2:**
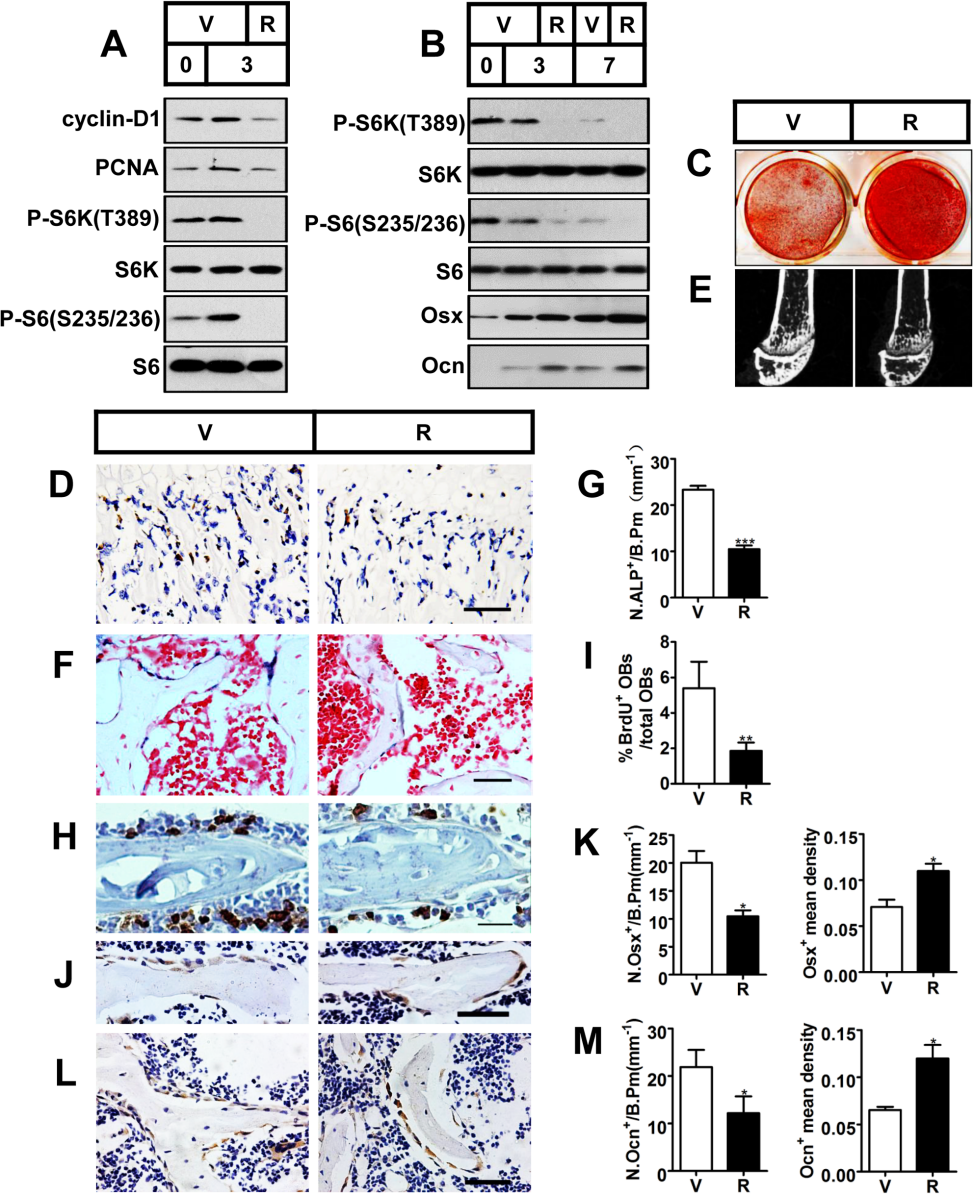
Inactivation of mTORC1 prevents preosteoblast proliferation but enhances their differentiation *in vitro* and *in vivo*. (A) Proliferating MC3T3-E1 cells were treated with vehicle (V) or 0.1 nM rapamycin (R) and underwent immunoblotting to detect proliferative markers (cyclin-D1 and PCNA) on the day of cell plating (day 0) and on the 3^rd^ day. (B) Differentiating MC3T3-E1 cells were treated with vehicle or 0.1 nM rapamycin for the indicated time (3d, 7d) and underwent immunoblotting to detect osteoblastic markers (Osx and Ocn). (C) AR-S staining of differentiated MC3T3-E1 cells on the 14^th^ day of osteogenic induction. (D) Immunohistochemistry staining for S6 phosphorylation (Ser235/236) in sections of distal femur of 10-week-old female C57BL/6 mice treated with vehicle (V) or rapamycin (R). (E) Micro-CT images of metaphyseal trabecular bone of the distal femur. (F) Alkaline phosphatase (ALP) staining of trabecular bone in sections from the distal femora (G) Quantification of osteoblast lineage cells stained with ALP. N.ALP^+^/B.Pm, number of ALP positive cells per bone perimeter (mm^-1^). Representative immunohistochemistry staining for (H) BrdU, (J) osterix, and (L) osteocalcin in femur sections. (I) Percentage of BrdU^+^ osteoblasts out of total osteoblasts on bone surface. (K, M) Number of Osx-positive cells (N.Osx^+^) and Ocn-positive cells (N.Ocn^+^) on the bone surface was measured as cells per millimeter of perimeter in sections (/B.Pm) and mean density of the corresponding positive cells was calculated as integrated optical density (IOD) per area of positive cells. All data are mean ± SD (n = 3 mice).Scale bar, 100 μm for (D) and 50 μm for (F), (H), (J), (L). *P < 0.05, **P < 0.01, ***P<0.001 by t test.

We next investigated the role of reduced mTORC1 activity caused by rapamycin in the proliferation and differentiation of preosteoblasts. As shown in Fig 1A, the growth of cells treated with 0.1 nM rapamycin significantly lagged behind control cells, and the decreased level of proliferative markers (cyclin D1 and PCNA) in rapamycin-treated cells revealed the underlying mechanism (Fig 2A). We next determined the role of reduced mTORC1 activity in the differentiation of preosteoblasts. As seen in Fig 2B, a low concentration of rapamycin (0.1nM) increased the expression of osterix and osteocalcin. Separate sets of cells were tested for mineralization capacity, a terminal differentiation parameter for osteoblasts, by staining with alizarin red, and the results confirmed enhanced mineralization of the extracellular matrix (ECM) in MC3T3-E1 cells with impaired mTORC1 activity (Fig 2C). Increased mineralization of ECM was also observed in fetal rat calvarial cells treated with 0.1nM rapamycin (S1 Fig).

To test the results *in vivo*, we subcutaneously injected newborn wild-type C57BL/6 mice with rapamycin daily (0.1 mg/kg body weight/day) for 10 weeks. mTORC1 activity was efficiently down-regulated in the primary spongiosa as observed by the reduction in immunohistochemistry staining for S6 phosphorylation (Ser235/236) in rapamycin-treated mice (Fig 2D). Micro-CT analysis of the distal femur showed a marked decrease in bone mass in the rapamycin-treated mice (Fig 2E), as demonstrated by a significant decrease in BV/TV, trabecular number or trabecular thickness, coupled with an increase in trabecular separation (Table 1). In line with the reduced trabecular bone mass, mice treated with rapamycin showed decreased cortical thickness as well as smaller outer and inner femoral mid-shaft bone perimeters when compared with controls (Table 1). The decrease in cortical thickness was due to deficient periosteal apposition, as evidenced by a decrease in the outer perimeter of the mid-shaft, while the inner perimeter was also decreased.

**Table 1:** Micro CT analysis of rapamycin-treated C57BL/6 mice at 10 weeks of age.

Parameters	Vehicle	RAPA	RAPA/Vehicle	P value
**Cancellous bone**				
BMD[mg HA/ccm]	718.98±13.51	653.54±13.12	0.9	0.008389
BV/TV[1]	0.21±0.01	0.09±0.02	0.4	0.003005
Tb.N[1/mm]	5.60±0.41	2.32±0.20	0.4	0.000102
Tb.Sp[mm]	0.18±0.01	0.46±0.04	2.6	0.000178
Tb.Th[mm]	0.061±0.003	0.042±0.004	0.7	0.006392
**Cortical bone**				
BMD[mg HA/ccm]	966.24±3.24	917.50±6.88	0.9	0.000207
Ct.Th[mm]	0.212±0.015	0.162±0.003	0.8	0.013096
outer perimeter[mm]	5.93±0.33	4.07±0.20	0.7	0.001464
inner perimeter[mm]	4.41±0.47	3.27±0.23	0.7	0.059742

RAPA: rapamycin

BMD: bone mineral density

BV: bone volume

TV: total volume

Tb.N: trabecular number

Tb.Sp: trabecular separation

Tb.Th: trabecular thickness

Ct.Th: cortical bone thickness.

Values are shown as mean±SD (n = 5).

We then analyzed the cellular basis of decreased bone mass in rapamycin-treated mice. Tartrate-resistant acid phosphatase (TRAP) staining on femoral sections revealed a reduction in osteoclasts within the trabecular bone region in rapamycin-treated mice (S2 Fig). Thus, decreased bone mass in the rapamycin-treated mice was not caused by an overall decrease in bone resorption. We next investigated bone formation parameters by first examining the total number of osteoblasts. ALP staining of the distal femoral section from rapamycin-treated mice showed a marked reduction in osteoblastic lineage cells when normalized to the bone perimeter (Fig 2F and 2G). Immunohistochemistry staining for BrdU revealed that proliferation of osteoblastic lineage cells was attenuated in rapamycin-treated mice (Fig 2H and 2I). To determine if differentiation of osteoblasts was affected, we next measured their numbers at different stages of differentiation by immunostaining femur sections from rapamycin-treated mice and controls. The number of osterix-positive preosteoblasts (Fig 2J and 2K) and osteocalcin-positive mature osteoblasts (Fig 2L and 2M) on the bone surfaces of rapamycin-treated mice were less than those in the controls. However, the mean density of osterix and osteocalcin-positive cells was increased in mice treated with rapamycin (Fig 2K and 2M), indicating that expression of osterix and osteocalcin in single osteoblasts was increased in rapamycin-treated mice. Thus, in agreement with the *in vitro* results, rapamycin attenuated proliferation of preosteoblasts but promoted their differentiation *in vivo*.

### mTORC1 activation in preosteoblasts produces immature woven bone

**Fig 3:**
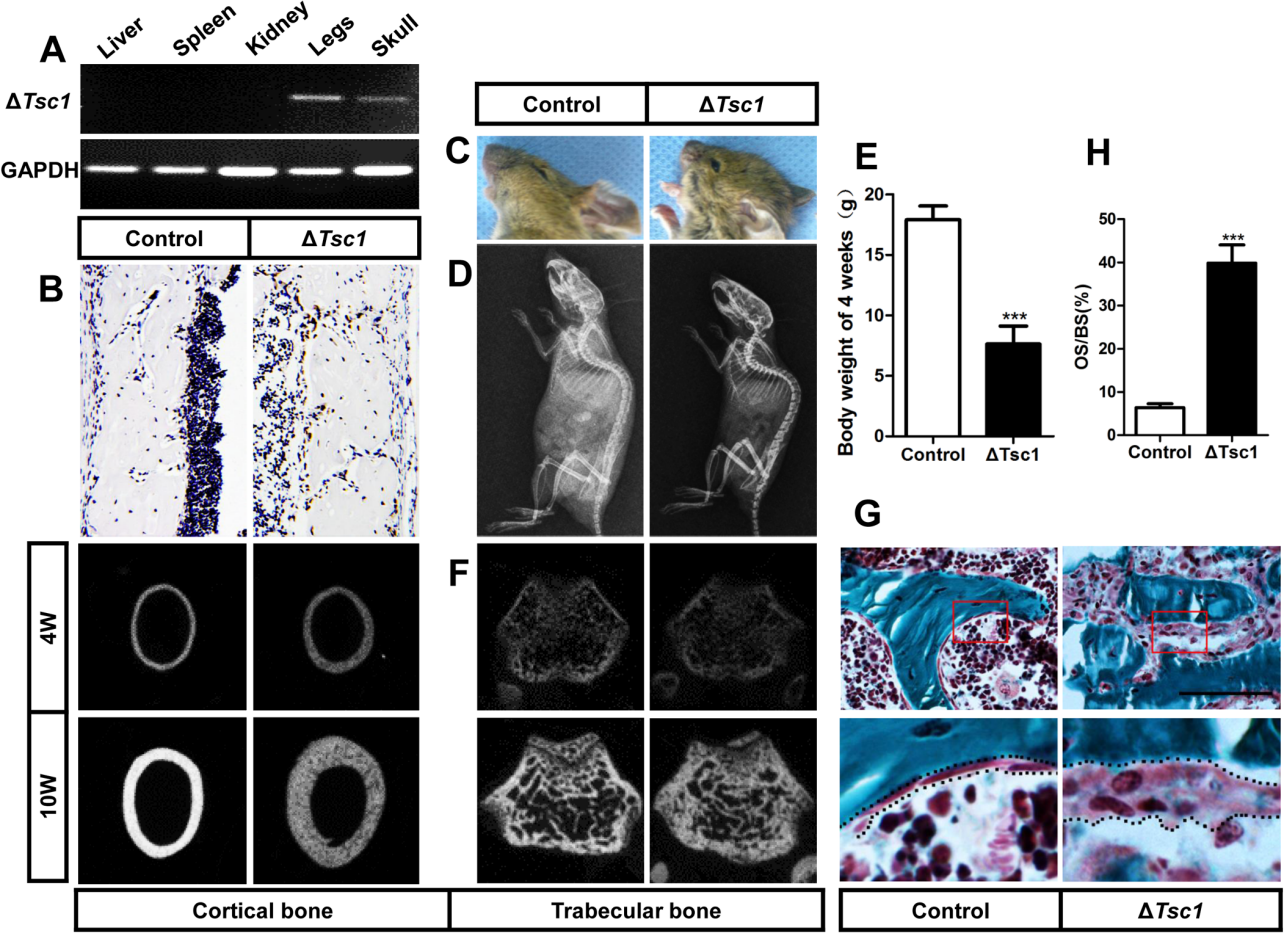
mTORC1 activation in preosteoblasts produces immature woven bone. (A) PCR analysis of *Tsc1* allele recombination in tissues from *Osx*-*GFP*::*Cre* ^TG/+^;*Tsc1* ^flox/flox^ mice (Δ*Tsc1* mice). Primers for *GAPDH* were used as a loading control. (B) Immunohistochemical staining of S6 phosphorylation (Ser235/236) in sections of distal femur from 10-week-old control and Δ*Tsc1* mice. (C) The 10-week-old Δ*Tsc1* mouse had a square skull (the result of hyperplasia of the osteoid, a typical manifestation of rickets) compared with the control littermate. (D) Plain X-ray examination of a 4-week-old Δ*Tsc1* mouse revealed a smaller skeleton and higher bone mass compared with the control littermate. (E) Body weight of 4-week-old control and Δ*Tsc1* mice. (F) Representative images of micro-CT analyses of the structure of metaphyseal trabecular bone and cortical bone in the distal femur showed increased bone mass, but more porous areas (indicating more hypomineralized areas) in Δ*Tsc1* mice. (G, H) Goldner’s Masson trichrome staining of distal femur showed more osteoid/hypomineralized areas (stained red) in bone of Δ*Tsc1* mice. Boxed area is enlarged in the panel below. The dotted line marked boundary of the red osteoid. OS/BS, osteoid per bone surface. Scale bar, 100μm.

To characterize the role of mTORC1 activation in the regulation of proliferation and differentiation of osteogenic progenitors, we generated mice in which mTORC1 were selectively activated in osteoprogenitor cells committed to the osteoblast lineage. To achieve such cell type-specific knockout, we crossed floxed *Tsc1* mice with *Osx*-*GFP*::*Cre* mice (which express a GFP-Cre fusion protein under the direction of the *Osx1* promoter) to generate conditional *Tsc1* knockout mice. We mated *Osx*-*GFP*::*Cre* ^TG/+^;*Tsc1* ^flox/+^ mice and selected female mice with the genotype *Osx-GFP*::*Cre* ^TG/+^;*Tsc1* ^flox/flox^ (hereafter, referred to as Δ*Tsc1*) for detailed analysis. Female *Osx*-*GFP*::*Cre* ^TG/+^;*Tsc1* ^+/+^ littermates served as controls. Δ*Tsc1* mice were born at the expected Mendelian frequency, and recombination of *Tsc1* alleles only occurred in skeletal tissues (i.e., legs and skull) as demonstrated by allele specific PCR (Fig 3A). Immunohistochemical staining of distal femur sections showed a dramatic increase in S6 phosphorylation (Ser235/236) in Δ*Tsc1* mice (Fig 3B), indicating that mTORC1 was activated by *Tsc1* disruption.

At the gross level, 4-week-old Δ*Tsc1* mice demonstrated square skulls and prominent dwarfism (Fig 3C and 3D). The body weight of the *Tsc1*-deficient mice was significantly lower than that of their control littermates, indicating retardation of growth in the mutant mice (Fig 3E). Whole body X-ray analysis showed increased bone mass in the skull, vertebrae and long bones (Fig 3D). Because of thickened cortical bone, pale bones were observed in Δ*Tsc1* mice (S3 Fig). Micro-CT analysis of the distal regions of the femur confirmed a marked increase in cancellous bone mass in Δ*Tsc1* mice (Fig 3F), as reflected in a 240%, 140% and 170% increase in BV/TV, trabecular number and trabecular thickness, respectively, coupled with a 50% decrease in trabecular separation. Analysis of femoral mid-shaft revealed a similar increase in cortical bone mass in Δ*Tsc1* mice, as reflected in a 230% and 130% increase in cortical thickness and outer perimeter, respectively, and a 90% decrease in inner perimeter (Table 2). Although bone mass was increased in Δ*Tsc1* mice, micro-CT radiograms showed more porous areas in the bones of mutant mice (Fig 3F), which resulted in a uniform 90% decrease in bone mineral density (BMD) in cancellous and cortical bone (Table 2). The appearance of more porous areas in mutant mice was the result of increased areas of hypomineralization. As shown by Goldner’s Masson trichrome staining, Δ*Tsc1* mice had 35% more osteoid/hypomineralized areas (stained red) in bone (Fig 3G and 3H). Together, these data suggest that mTORC1 activation in osteogenic progenitors stimulates these progenitors to produce increased amounts of immature woven bone.

**Table 2:** Micro CT analysis of ΔTsc1 mice at 10 weeks of age.

Parameters	Control	Δ*Tsc1*	Δ*Tsc1*/ Control	P value
**Cancellous bone**				
BMD[mg HA/ccm]	713.56 ± 13.18	660.81 ± 16.08	0.9	0.034874
BV/TV[1]	0.14 ± 0.03	0.40 ± 0.04	2.4	0.000690
Tb.N[1/mm]	4.05 ± 0.51	5.84 ± 0.16	1.4	0.010364
Tb.Sp[mm]	0.29 ± 0.05	0.14 ± 0.01	0.5	0.017358
Tb.Th[mm]	0.050 ± 0.002	0.096 ± 0.006	1.7	0.000063
**Cortical bone**				
BMD[mg HA/ccm]	941.78 ± 12.37	880.22 ± 10.76	0.9	0.005584
Ct.Th[mm]	0.181 ± 0.010	0.417 ± 0.010	2.3	0.000000
outer perimeter[mm]	4.78 ± 0.19	5.99 ± 0.16	1.3	0.001270
inner perimeter[mm]	3.78 ± 0.17	3.33 ± 0.07	0.9	0.039561

Values are shown as mean±SD (n = 5)

### mTORC1 activation promotes preosteoblast proliferation but prevents their differentiation

**Fig 4:**
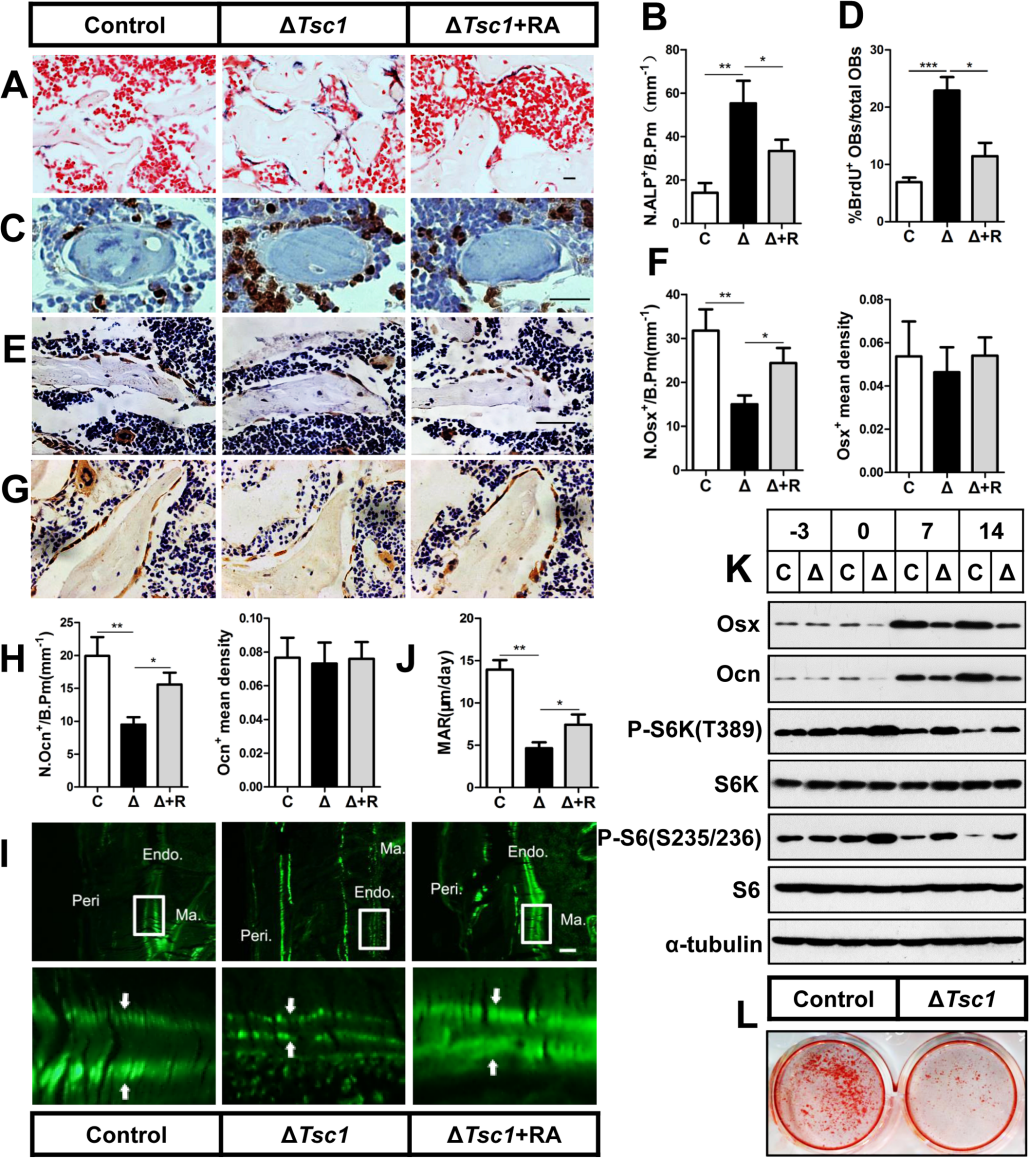
mTORC1 promotes proliferation of preosteoblasts but prevents their maturation. (A) ALP staining in sections of distal femur from 10-week-old control (C), Δ*Tsc1* (Δ) and rapamycin treated Δ*Tsc1* (Δ+R) mice. (B) Number of ALP positive cells per bone perimeter (mm^-1^) (N.ALP^+^/B.Pm). Immunohistochemistry staining for (C) BrdU, (E) osterix (Osx) and (G) osteocalcin (Ocn) in distal femur. (D) Percentage of BrdU^+^ osteoblasts out of total osteoblasts on bone surface. (F, H) Number of osterix positive cells (N.Osx^+^) and osteocalcin positive (N.Ocn^+^) on the bone surface was measured as cells per millimeter of perimeter in sections (/B.Pm) and the mean density of the corresponding positive cells was calculated as integrated optical density (IOD) per area of positive cells. (I) Calcin double labeling of cortical bone in distal femora. Boxed area is enlarged in the panel below. Ma.: marrow, Endo.: endosteum, Peri.: periosteum. (J) Mineral apposition rate (MAR). (K) Western blot analysis of differentiating primary calvarial preosteoblasts showed decreased expression of osterix and osteocalcin following doxycycline discontinuation in Δ*Tsc1* cells (0, 7^th^, 14^th^ day). (L) Alizarin red staining of differentiated primary calvarial preosteoblasts on the 14^th^ day showed decreased mineralized nodules in Δ*Tsc1* cells. All data are mean ± SD (n = 5 mice), scale bars represent 50 μm for (A), (C), (E), (G) and 100 μm for (I). *P < 0.05, ** P < 0.01, ***P<0.001 by t test.

We then analyzed the cellular basis for the increased amounts of immature woven bone in Δ*Tsc1* mice. ALP staining of 10-week-old Δ*Tsc1* mouse femurs indicated an increased number of osteoblast lineage cells (Fig 4A and 4B). To determine the underlying molecular mechanism of the increase in osteoblasts in transgenic bone, we cultured postnatal day 3 calvarial osteoblasts and found a significant increase in BrdU-positive cells, indicating increased cellular proliferation in Δ*Tsc1* calvarial osteoblasts (S4 Fig). In addition, the percentage of proliferative osteoblasts on bone surface was increased in Δ*Tsc1* mice (Fig 4C and 4D).

We next determined whether the differentiation of preosteoblasts was influenced in Δ*Tsc1* mice. Osteoblasts in Δ*Tsc1* mice lost normal morphology and appeared immature as shown by scanning electron microscopy (SEM) analyses (S5 Fig), indicating a faulty maturation process from precursors to osteoblasts. Immunohistochemical staining of distal femur sections showed decreased expression of osterix (Fig 4E and 4F) and osteocalcin (Fig 4G and 4H), indicating impaired differentiation of preosteoblasts in mutant mice. To detect osteoblast activity, we performed double fluorochrome labeling analyses. Incorporation of the two fluorochromes was evident in the control mice bone, while mutants’ bone displayed diffuse fluorochrome labeling, a characteristic feature of immature woven bone. Although mineralizing surface was dramatically increased abnormally in cortical bone, distance between calcin-labeled mineralization fronts at endosteum of the midshaft of femur was smaller in Δ*Tsc1* mice than that in the controls (Fig 4I). Histomorphometric measurements showed that the endosteum mineral apposition rate (MAR) of Δ*Tsc1* mice was lower than that of controls (Fig 4J), suggesting impaired performance of individual osteoblasts in Δ*Tsc1* mice.

To further examine the impact of mTORC1 activation on osteoblastic precursor differentiation, we used primary calvarial preosteoblast cultures in which *Tsc1* was eliminated prior to the induction of osteoblast differentiation due to suppression of *Osx-Cre* by doxycycline to specifically delete *Tsc1*. On the day the cells reached confluence (day -3), mTORC1 was not activated (as indicated by unchanged phosphorylation of S6K and S6) and markers of differentiated osteoblasts (Osx and Ocn) remained normal in Δ*Tsc1* cells compared with the controls. Doxycycline was then discontinued to activate *Osx-Cre* and delete *Tsc1* and osteoblastic differentiation was induced for another 14 days. Total proteins were extracted for western blot on different days after induction (day 0, 7, 14). Osteoblasts lacking *Tsc1* exhibited the expected activation of mTORC1 and reduced expression of osterix and osteocalcin (Fig 4K). Alizarin red staining confirmed that ΔTsc1 mice exhibited a marked decreased capacity to form mineralized nodules (Fig 4L).

Notably, osteoclast numbers on the bone surface (OC.N/B.Pm) were significantly decreased in mutant mice (S6 Fig).These data suggest that mTORC1 activation in preosteoblasts promotes their proliferation but prevents their differentiation and decreases osteoclast number by an undefined mechanism.

### Rapamycin reverses phenotypes in Δ*Tsc1* mice

**Fig 5:**
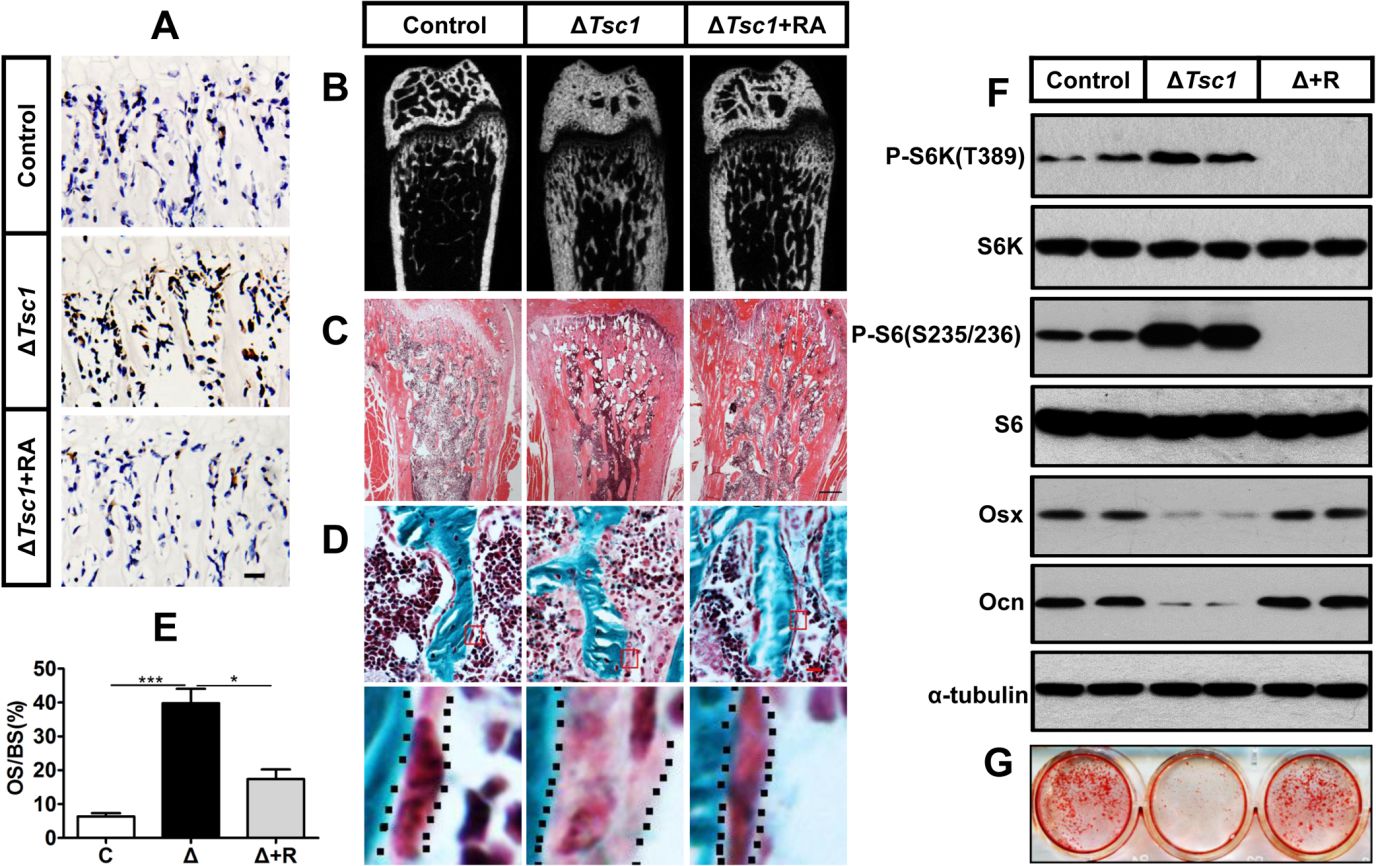
Rapamycin reverses phenotypes in Δ*Tsc1* mice. (A) Immunohistochemical staining of phosphorylation of S6 (Ser235/236) in sections of distal femur from 10-week-old control, Δ*Tsc1* and rapamycin treated Δ*Tsc1* mice. (B) Representative images of micro-CT analyses in the distal femur showed reduced bone mass in rapamycin treated Δ*Tsc1* mice. (C) HE staining of distal femur showed expanded bone marrow cavity in rapamycin treated Δ*Tsc1* mice. (D, E) Goldner’s Masson trichrome staining showed reduced osteoid in rapamycin treated Δ*Tsc1* mice. OS/BS, osteoid per bone surface. Scale bar, 100 μm. ***P < 0.001, * P < 0.05 by t test. (F) Western blot analysis showed elevated osterix and osteocalcin expression in Δ*Tsc1* calvarial cells treated with rapamycin (0.1nM). (G) Alizarin red staining of differentiated primary calvarial preosteoblasts on the 14^th^ day showed increased mineralized nodules in rapamycin treated Δ*Tsc1* cells.

To identify whether the increased amount of immature woven bone was mTORC1-dependent, Δ*Tsc1* mice were administered with rapamycin prenatally from E13.5 (the approximate day of *Osx-Cre* expression) and then after birth until 10 weeks old. After treatment with 0.1 mg/kg/day rapamycin, S6 phosphorylation (Ser235/236) was significantly decreased in the primary spongiosa (Fig 5A), accelerated proliferation was reduced to normal level (Fig 4C and 4D), increased osteoblastic lineage cells with ALP (Fig 4A and 4B) were reduced to normal, expression of osterix (Fig 4E and 4F) and osteocalcin (Fig 4G and 4H) was increased, diffuse and narrowed distance between fluorochrome labeling was distinct and broadened (Fig 4I and 4J), high bone mass was notably reduced (Fig 5B), narrowed bone marrow cavity was expanded (Fig 5C), areas of hypomineralization were decreased to normal (Fig 5D and 5E) and bone architecture was partially normalized (Table 3). These results indicate that preosteoblasts in Δ*Tsc1* mice re-acquired the ability to differentiate, and form normal bone. The results of rapamycin treatment in differentiating calvarial cells confirmed these findings. Following treatment with a low concentration (0.1 nM) of rapamycin for 14 days, decreased expression of osterix and osteocalcin was elevated (Fig 5F) and mineralized nodules were formed increasingly (Fig 5G) in Δ*Tsc1* cells.

**Table 3:** Micro CT analysis of rapamycin-treated ΔTsc1 mice at 10 weeks of age.

Parameters	Δ*Tsc1*	Δ*Tsc1+*RA	Δ*Tsc1+*R A/ Δ*Tsc1*	P value
**Cancellous bone**				
BMD[mg HA/ccm]	660.81 ± 16.08	710.31 ± 4.47	1.1	0.017986
BV/TV[1]	0.40 ± 0.04	0.26 ± 0.04	0.6	0.045705
Tb.N[1/mm]	5.84 ± 0.16	6.21± 0.34	1.2	0.354591
Tb.Sp[mm]	0.14 ± 0.01	0.28 ± 0.03	1.9	0.003653
Tb.Th[mm]	0.096 ± 0.006	0.062 ± 0.004	0.6	0.001050
**Cortical bone**				
BMD[mg HA/ccm]	880.22 ± 10.76	947.25 ± 5.46	1.1	0.000536
Ct.Th[mm]	0.417 ± 0.010	0.259 ± 0.027	0.7	0.000555
outer perimeter[mm]	5.99 ± 0.16	4.95 ± 0.16	0.9	0.001926
inner perimeter[mm]	3.33 ± 0.07	3.35 ± 0.05	1.0	0.780548

Values are shown as mean±SD (n = 5).

### mTORC1 inhibits Runx2 expression by activating Notch signaling in preosteoblasts

**Fig 6:**
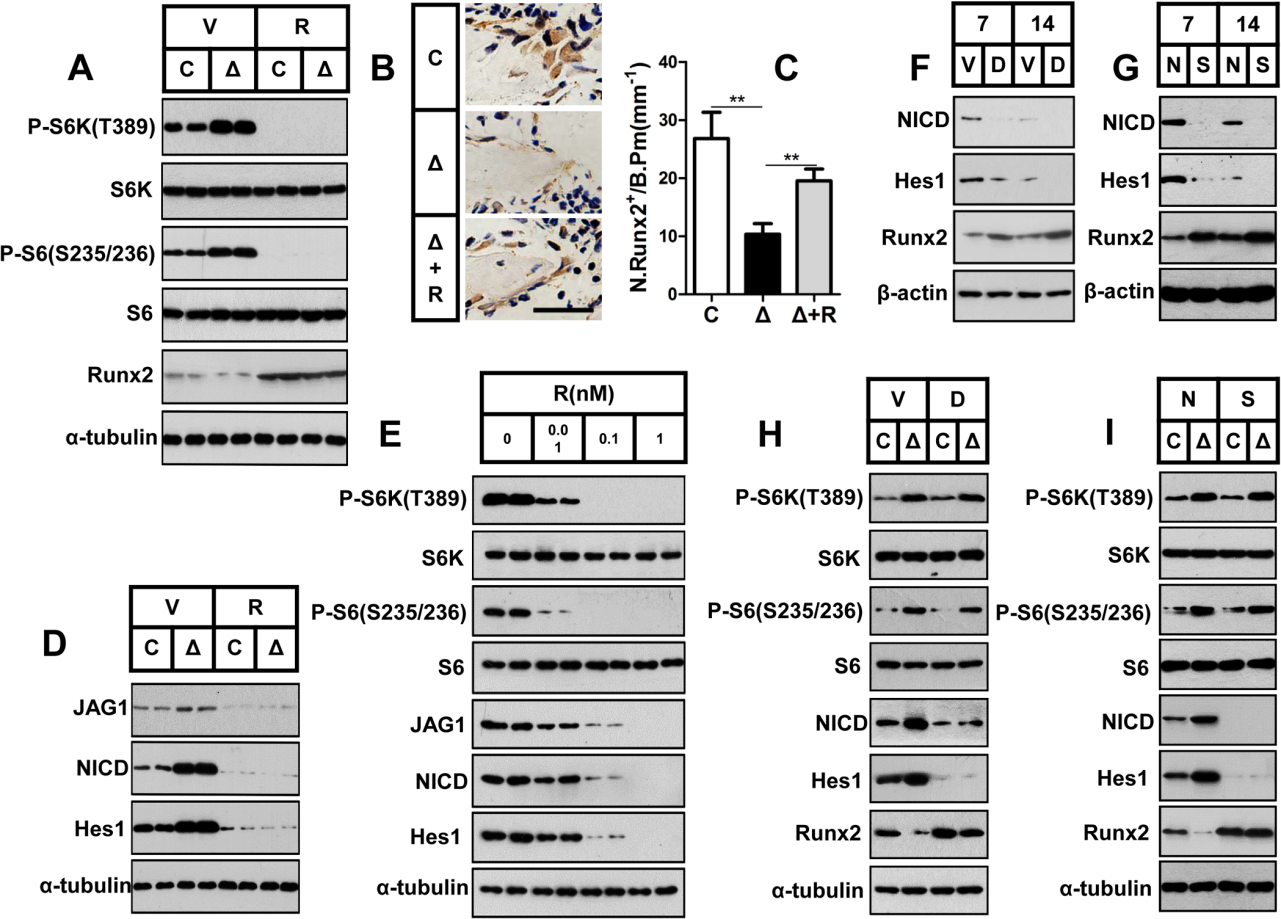
mTORC1 inhibits Runx2 expression by activating Notch signaling in preosteoblasts. (A) Control (C) and Δ*Tsc1* (Δ) primary calvarial cells were treated with vehicle (V) or 0.1 nM rapamycin (R) for 24 hours and then underwent immunoblotting to detect Runx2. (B) Immunohistochemical staining of Runx2 in sections of distal femur from 10-week-old control (C), Δ*Tsc1* (Δ) and rapamycin treated Δ*Tsc1* (Δ+R) mice. Scale bar, 50μm. (C) Number of Runx2 positive cells per bone perimeter (mm^-1^) (N.Runx2^+^/B.Pm).** P < 0.01 by t test. (D) Deletion of *Tsc1* caused elevated expression of Jagged1, NICD and Hes1, while treatment with 0.1 nM rapamycin for 24 hours led to decreases in these parameters in primary calvarial cells. (E) Rapamycin dose-dependently reduced the expression of Jagged1, NICD and Hes1 in MC3T3-E1 cells. Differentiating MC3T3-E1 (F, G) and primary cavarial cells from control and Δ*Tsc1* mice (H, I) were treated with 50 μM DAPT (F, H) or *Notch1* siRNA (G, I) and then underwent immunoblotting to detect Runx2. V: vehicle; D: DAPT; N: negative control; S: si-*Notch1*.

We next investigated the mechanism by which mTORC1 regulates osteoblast differentiation. Runx2, a Runt domain-containing transcription factor, is required for commitment of mesenchymal osteochondroprogenitors to the osteoblastic lineage, differentiation into mature osteoblasts and terminal differentiation into osteocytes [32]. Δ*Tsc1* calvarial cells exhibited a reduced Runx2 expression level, while rapamycin treatment led to an increase in Runx2 above baseline (Fig 6A). The regulation of Runx2 by mTORC1 was further confirmed in Δ*Tsc1* mice (Fig 6B and 6C). Thus, mTORC1 attenuated the expression of Runx2 *in vitro* and *in vivo*, and the delay in osteoblast differentiation was probably due to repression of Runx2 in Δ*Tsc1* mice.

In consideration of the same negative role of Notch signaling in osteoblast differentiation [32,33,34,35] as mTORC1 signaling and the positive correlation between Notch and mTORC1[28,29,30], we next investigated the potential role of the Notch pathway in the mechanism underlying the impaired differentiation potential of mTORC1-activated cells. Jagged1 (a Notch ligand) protein expression was significantly enhanced in primary Δ*Tsc1* calvarial cells, and rapamycin reduced this expression to normal (Fig 6D) and dose-dependently decreased its expression in MC3T3-E1 cells (Fig 6E). In addition, a similar pattern of expression of the Notch transactivator NICD domain and Hes1 (a direct target of Notch) was also observed in these cells (Fig 6D and 6E). These results demonstrated that mTORC1 activated Notch signaling in preosteoblasts.

As the Notch pathway has been reported to reduce Runx2 activity [32], we next determined whether Notch signaling impaired differentiation of preosteoblasts upstream of Runx2. We found increased Runx2 expression in differentiating MC3T3-E1 and primary cavarial cells following suppression of the Notch pathway using the Notch inhibitor N-[N-(3, 5-difluorophenacetyl-L-alanyl)]-S-phenylglycinet-butylester (DAPT) (Fig 6F and 6H) and Notch siRNA (Fig 6G and 6I). We conclude that mTORC1 inhibited Runx2 expression by activating Notch signaling in preosteoblasts.

### mTORC1 activates Notch pathway in preosteoblasts through the STAT3/p63/Jagged cascade

**Fig 7:**
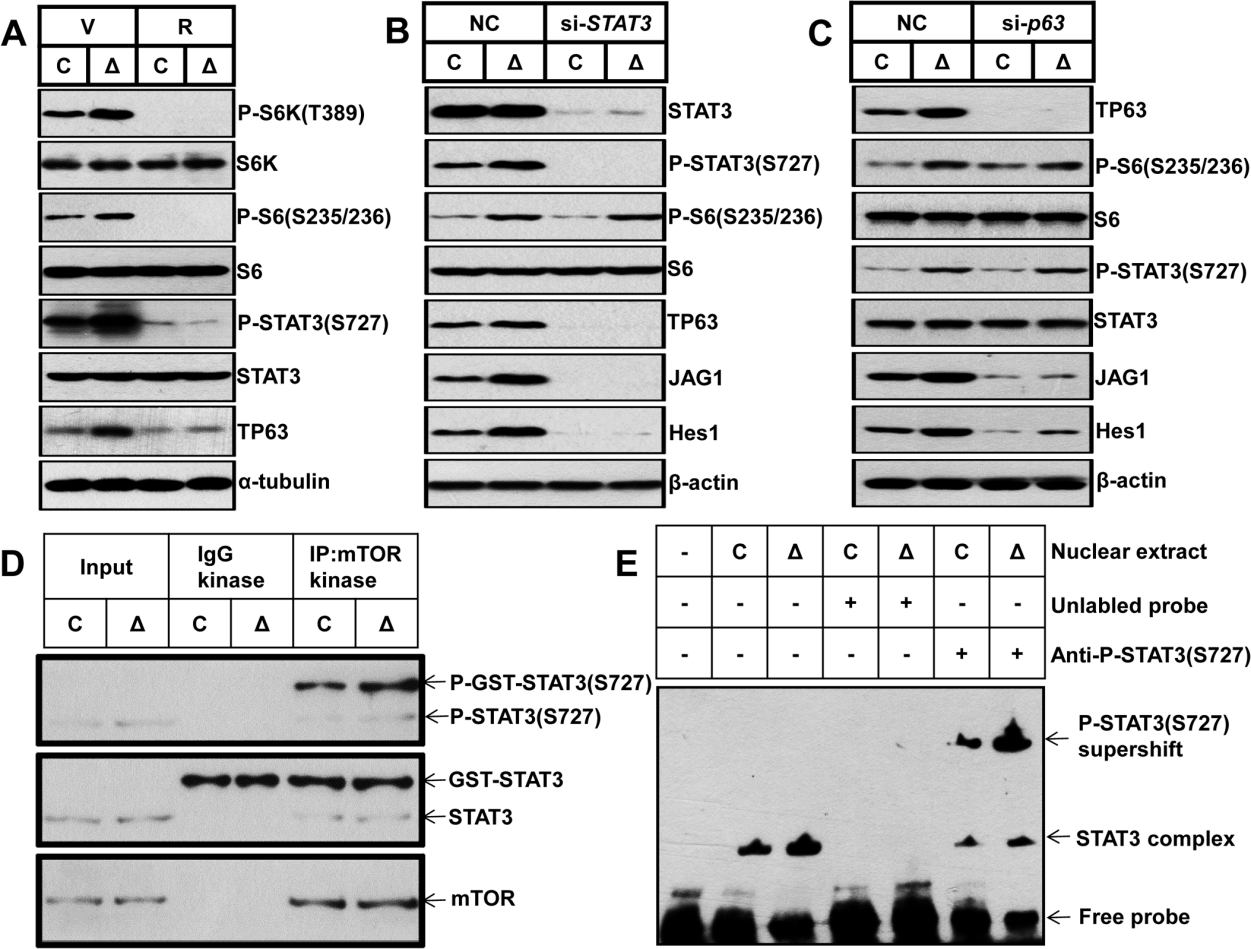
mTORC1 impairs osteoblast differentiation through the STAT3/p63/Jagged/Notch cascade upstream of Runx2. (A) Control and Δ*Tsc1* primary calvarial cells were treated with vehicle (V) or 0.1 nM rapamycin (R) for 24 hours and then underwent immunoblotting to detect phosphorylation of STAT3 (Ser727) and TP63 (total p63). Control and Δ*Tsc1* primary calvarial cells were treated with *STAT3* (B) and *p63* (C) siRNA and negative control (NC) for 48 hours and then underwent immunoblotting to detect mTORC1 activity, phosphorylation of STAT3(Ser727), TP63 (total p63) and activity of Notch pathway (JAG1, Hes1). (D) Cultured primary calvarial cells were immunoprecipitated with anti-mTOR antibody and the precipitated mTOR was assayed for kinase activity against recombinant GST-tagged full-length STAT3. (E) Nuclear extract from control and Δ*Tsc1* primary calvarial cells were analyzed using EMSA. Binding of STAT3 to biotin-labeled DNA probes is shown as “STAT3 complex”. To compete with the binding, an unlabeled STAT3 binding site DNA probe was added to the reaction in 200 times molar excess. Adding anti-pSTAT3 (S727) antibody to the reactions caused reduction of STAT3-DNA binding and bands of supershift.

To define how activated mTORC1 influenced the Jagged1/Notch/Hes1 pathway, we determined the expression of STAT3 and p63, as STAT3 is a transcriptional activator of p63 [36] as well as the downstream target of mTOR [37], and p63 is a positive regulator of Jagged expression and Notch activity [38,39,40] which is induced by PI3K [41]. Cavarial cells with disruption of *Tsc1* showed increased phosphorylation of STAT3 on Ser727 and expression of TP63 (total p63) (Fig 7A), and rapamycin reduced these parameters to normal levels. Knockdown of *STAT3* by siRNA led to reduction in p63, Jagged1 and Hes1 without affecting the activity of mTORC1 (Fig 7B), and downregulation of *p63* resulted in reduced Jagged1 and Hes1 but no changes of STAT3 phosphorylation and mTORC1 activity (Fig 7C). These findings suggested that the STAT3/p63 cascade is positively regulated by mTORC1 and serves as a conjunction between mTORC1 and the Notch pathway.

We next sought to define how mTORC1 regulates STAT3/p63 cascade in osteoblasts. As has been reported that mTORC1 directly phosphorylates STAT3 at Ser727 [37], we firstly examined whether mTORC1 could phosphorylate STAT3 at Ser727 in osteoblasts. We immunoprecipitated mTOR complex from control and Δ*Tsc1* calvarial cells and subjected the immunoprecipitates to *in vitro* kinase assays using a recombinant GST-tagged full-length STAT3 peptide. As shown in Fig 7D, mTOR immunoprecipitates could phosphorylate STAT3 at Ser727 *in vitro* and constitutive activated mTORC1 from Δ*Tsc1* calvarial cells enhanced the phosphorylation. Thus the elevated phosphorylation of STAT3 (S727) in Δ*Tsc1* osteoblasts is due to increased mTOR kinase activity. Next, we examined the role of phosphorylated STAT3 (S727) in the regulation of the *p63* gene transcription by electrophoretic mobility shift assay (EMSA). Nuclear protein of osteoblasts bound specifically to a double-strand probe containing a consensus STAT3-specific binding sequence in the promoter region of *ΔNp63* [36], and anti-pSTAT3 (S727) antibody significantly reduced the binding of STAT3 to *p63* promoter (Fig 7E). Accordingly, nuclear protein from Δ*Tsc1* osteoblasts showed an increased binding of pSTAT3 (S727) to the probe (Fig 7E). Taken together, these results suggest that phosphorylated STAT3 (S727) may bind to *p63* promoter to regulate its transcription in osteoblast, and mTORC1 activates the Notch pathway through STAT3/p63/Jagged cascade.

### mTORC1 impairs preosteoblast differentiation through the Notch pathway upstream of Runx2

**Fig 8:**
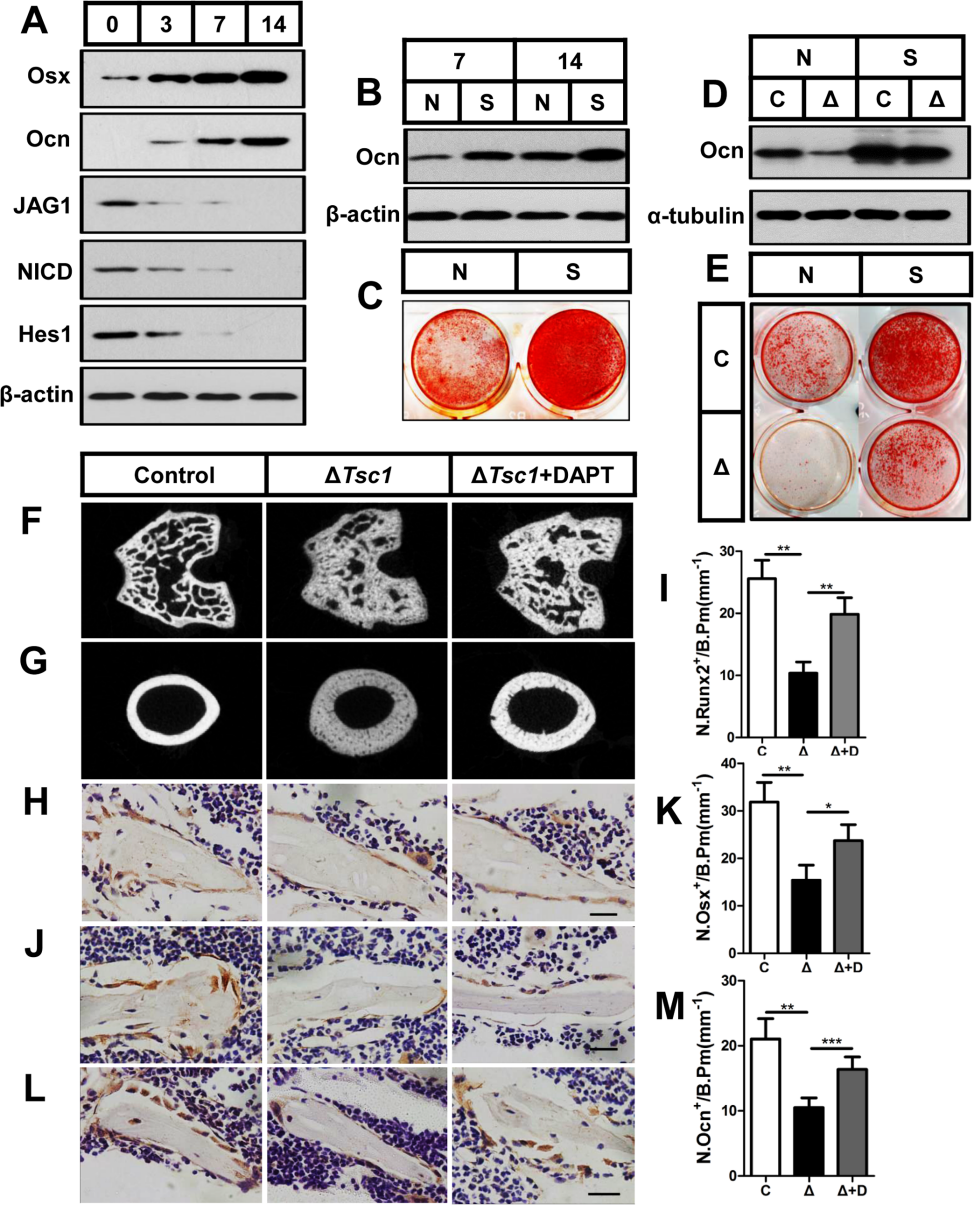
mTORC1 impairs osteoblast differentiation through the Notch pathway upstream of Runx2. (A) Western blot analysis of the expression of Jagged1, NICD and Hes1 during differentiation of MC3T3-E1 cells. Differentiating MC3T3-E1 cells were treated with *Notch1* siRNA (S) and then underwent immunoblotting to detect osteocalcin (B) on the 7^th^ and 14^th^ day and alizarin red staining (C) on the 14^th^ day. Differentiating control (C) and Δ*Tsc1* (Δ) primary calvarial cells were treated with negative control (N) or si-*Notch1* (S) and then underwent immunoblotting to detect osteocalcin (D) and alizarin red staining (E) on the 14^th^ day. 6-week old Δ*Tsc1* mice were administered with DAPT (10mg/kg per day) or equivalent volume of DMSO for 4 weeks, micro-CT images of trabecular bone in secondary ossification center (F) and cortical bone of midshaft (G) of femur were shown. Immunohistochemistry staining for Runx2 (H), osterix (Osx) (J), and osteocalcin (Ocn) (L) in distal femur. Numbers of Runx2 (N.Runx2^+^) (I), osterix (N.Osx^+^) (K) and osteocalcin (N.Ocn^+^) (M) positive cells on the bone surface were measured as cells per millimeter of perimeter in sections (/B.Pm). All data are mean ± SD (n = 5 mice), all scale bars represent 50 μm. *P < 0.05, ** P < 0.01, *** P < 0.001 by t test.

As mTOR is a positive regulator of the Jagged1/Notch/Hes1 pathway, we next determined whether hyperactive Notch signaling was responsible for the impaired differentiation of preosteoblasts by mTORC1 activation. Firstly, we examined the correlation between the level of Notch activity and differentiation of preosteoblasts. Low activity of Notch signaling is required during differentiation of preosteoblasts, as the expression of Jagged1, NICD and Hes1 was decreased in parallel with an increase in the markers of osteoblast differentiation (Fig 8A), and inhibition of the Notch pathway by DAPT or si-*Notch1* promoted osteocalcin expression (S7A Fig and Fig 8B) and mineralized nodules formation in MC3T3-E1 cells (S7B Fig and Fig 8C). Importantly, a reduction in Notch by DAPT (S7C and S7D Fig) or siRNA (Fig 8D and 8E) potentiated the differentiation of Δ*Tsc1* preosteoblasts.

To test these results *in vivo*, we administered 6-week old mice with DAPT or equivalent volume of DMSO for 4 weeks. Micro-CT analysis of the distal femur showed a marked normalizing of bone architecture in DAPT-treated Δ*Tsc1* mice (Fig 8F and 8G and Table 4) when compared to their DMSO-treated control. Inhibition of Notch pathway by DAPT elevated expression of Runx2 and subsequent osterix and osteocalcin in osteoblasts (Fig 8H–8M), and significantly reversed the bone phenotypes of Δ*Tsc1* mice (Table 4). Therefore, Notch acts as an inhibitor of osteoblast differentiation, while deregulated mTORC1 activation impairs preosteoblast differentiation through activation of the Notch signaling pathway. 

**Table 4:** Micro CT analysis of DAPT-treated ΔTsc1 mice at 10 weeks of age.

Parameters	Δ*Tsc1*	Δ*Tsc1*+DAPT	Δ*Tsc1*+DAPT/ Δ*Tsc1*	P value
**Cancellous bone**				
BMD[mg HA/ccm]	631.15 ± 12.03	736.60 ± 29.42	1.1	0.000075
BV/TV[1]	0.40 ± 0.08	0.24 ± 0.07	0.6	0.006941
Tb.N[1/mm]	5.67 ± 0.49	7.08± 0.57	1.2	0.002934
Tb.Sp[mm]	0.14 ± 0.02	0.30 ± 0.02	2.1	0.000049
Tb.Th[mm]	0.103 ± 0.020	0.056 ± 0.009	0.6	0.001460
**Cortical bone**				
BMD[mg HA/ccm]	872.22 ± 38.40	960.64 ± 15.31	1.1	0.001386
Ct.Th[mm]	0.417 ± 0.026	0.261± 0.029	0.6	0.000020
outer perimeter[mm]	6.05± 0.34	5.07 ± 0.58	0.8	0.011741
inner perimeter[mm]	3.12 ± 0.22	4.16 ± 0.41	1.3	0.001126

Values are shown as mean±SD(n = 5).

Together, these *in vitro* and *in vivo* loss- and gain-of-function studies lend support for a central role of mTORC1 signaling in regulating both proliferation and differentiation of preosteoblast during bone homeostasis (S8 Fig).

## Discussion

In this study, we defined the essential role of mTORC1 signaling in osteoblastgenesis. We observed a decline in mTORC1 activity during differentiation of preosteoblasts and enhancement of osteoblastic differentiation following inhibition of mTORC1 *in vitro* and *in vivo*. Using conditional knockout cell and mouse models, we further revealed that activation of mTORC1 prevented preosteoblast differentiation through activation of the Notch signaling pathway.

mTORC1 is ubiquitously expressed in all types of cells to regulate growth and metabolism. Proliferation in many types of cells is promoted by mTORC1, thus it is not surprising that the activity of mTORC1 positively correlated with the proliferative rate of preosteoblasts. However, it is interesting that mTORC1 activity resulted in a decline in the differentiation of preosteoblasts. We speculate that the decline in mTORC1 activity is due to differentiated osteoblasts. As osteoblasts differentiate, calcification of the extracellular matrix gradually increases, which generates hyperosmolarity and cellular stress. Coincidentally, this type of stimulus has been shown to inhibit mTORC1 [42].

The role of mTORC1 in bone formation was originally identified in various cell lines and animals treated with rapamycin, however, the results were controversial. Depending on the cell type, rapamycin either stimulates or inhibits osteoblastic differentiation. In rat osteosarcoma (ROS 17/2.8) cells, rapamycin inhibited proliferation, but promoted osteogenic differentiation [16]. In C2C12 cells, rapamycin potentiated the effect of BMP-2 inducing late markers of osteoblast differentiation [17]. In Human Embryonic Stem Cells, rapamycin induced the up-regulation of early osteogenic markers and further promoted the expression of late osteoblastic marker mRNA and/or proteins and mineralized bone nodule formation following induction for 2–3 weeks [43]. On the other hand, rapamycin has been shown to block osteogenic protein-1 induction of alkaline phosphatase activity in fetal rat calvarial cells [19] and reduce alkaline phosphatase activity, osteocalcin expression and the calcium content in mesenchymal stem cells [20]. In MC3T3-E1 subclone 4 (MC-4) cells and primary mouse bone marrow stromal cells (BMSCs), rapamycin inhibited osteoblast differentiation by targeting osteoblast proliferation and the early stage of osteoblast differentiation [21]. Although the reasons for the discrepant results are not readily apparent, one possible explanation is that the cells used in these reports were in various stages of osteoblastic differentiation and mTORC1 activity is stage-specifically required during the differentiation process. The results of the present study demonstrated that inhibition of mTORC1 is essential for preosteoblast differentiation. Another possible explanation for these contradictory results could be related to the treatment conditions including concentration and duration. Rapamycin at a concentration as low as 0.1 nM is effective in significantly suppressing mTORC1 activity [21], while higher concentrations and long-term treatment may produce a decrease in cell viability and growth, and inhibition of mTORC2. Thus, we used a low dose of rapamycin (0.1 nM) as a precaution against non-specific effects, and obtained reliable results that low mTORC1 activity is crucial for osteogenesis.

Previous results in animals administered rapamycin have also been inconsistent. Administration of rapamycin for 14 days resulted in no change in cancellous bone volume in rats [23], and after a longer duration of treatment (28 days), rats still lacked an osteopenic phenotype despite increased bone turnover [24]. However, mice treated with a similar concentration and duration of treatment showed a phenotype with less new bone formation and lower trabecular bone mass [25], due to a reduced number of osterix and osteocalcin positive cells coupled with unaffected osteoclasts. In the present study, rapamycin-treated mice had a similar phenotype of bone loss as well as decreased numbers of osterix and osteocalcin positive cells, except that the mean density of these cells was enhanced and the number of osteoclasts was reduced by rapamycin. Although we could not rule out that the systemic effect of rapamycin acted on other cell types to affect bone mass indirectly, the results of rapamycin administration *in vivo* are in agreement with those from rapamycin treated cells *in vitro*, which consolidates our conclusions that hyperactive mTORC1 is not required for differentiation of preosteoblasts and rapamycin promotes preosteoblast differentiation by blocking mTORC1.

Clinically, rapamycin was initially identified as a macrocyclic antifungal agent [44] and is used for immunosuppression in transplantation. The experimental and clinical trials have showed that rapamycin reduced brain, kidney, and skin lesions of tuberous sclerosis complex (TSC) [45, 46]. Some TSC children are being started on rapamycin at a young age (< 5 years), and it is sometimes continued for many (>5) years [47, 48]. In this situation, bone densitometry and morphological measurements must be advised to monitor the possible side effects of rapamycin on bone. Rapamycin is likely to impair bone microarchitecture and quality of those children as indicated by the current study, although it presented no effect on growth in weight and height of them [48].

Recent mouse genetic studies also highlighted the critical role of mTORC1 in skeletal development, whereas the function of mTORC1 in osteoblast formation is uncertain and the underlying mechanisms are not defined. Mice with *Pten* [49] and *Tsc2* [50] disrupted by OC (osteocalcin)-cre recombinase (Δ*Tsc2*) in mature osteoblasts shared an elevated mTORC1 activity, and exhibited different phenotypes. Although both mouse models had uniform high bone mass, osteoblasts with deletion of *Pten* showed increased osteoblastic differentiation, while *Tsc2* disruption led to impaired differentiation of osteoblasts and mineralized nodule formation. Because inhibition of mTORC1 by rapamycin restored the differentiation defects in osteoblasts due to disruption of *Tsc2*, we reason that impairing osteoblastic differentiation is the function of hyperactive mTORC1 in mature osteoblasts, while an mTORC1-independent mechanism may account for the distinct phenotype in mice with disruption of *Pten*. Akt, which has been reported to promote osteoblast differentiation and bone development [51], is highly activated following *Pten* deletion and significantly inhibited following *Tsc2* disruption. Acceleration of osteoblast differentiation by Akt may exceed the inhibition due to mTORC1 activation and cause increased differentiation in osteoblasts with *Pten* ablation. Δ*Tsc2* mice presented a similar phenotype and same differentiation defect in osteoblasts as Δ*Tsc1* mice in the current study. However, in contrast to Δ*Tsc1* mice, Δ*Tsc2* mice exhibited a decreased proliferation rate of osteoblast, which indicates that mTORC1 may play different role in regulating proliferation of immature and mature osteoblasts, since OC (osteocalcin) is expressed exclusively in mature osteoblasts. Moreover, though mTORC1 activation resulted in a same defect of differentiation in mature osteoblasts and preosteoblasts, the underlying mechanisms may be different. In this sense, our work revealed the role of mTORC1 in preosteoblast differentiation under physiological and pathological conditions and explored a different mechanism (STAT3/p63/Jagged/Notch pathway) responsible. More recently, another similar phenotype of immature woven bone as that of Δ*Tsc1* mice has been reported in mice with disruption of *Lkb1* in preosteoblasts (Δ*Lkb1*) [52], but the increased trabecular bone mass was more severe in Δ*Lkb1* mice than in Δ*Tsc1* mice, as BV/TV increased 7-fold versus 2.4-fold. In contrast, cortical thickness, which showed a 2.3-fold increase in Δ*Tsc1* mice, was decreased 3-fold in Δ*Lkb1* mice and osteoclasts were increased inversely in Δ*Lkb1* mice when compared with Δ*Tsc1* mice. These discrepancies may also be attributed to the activation of mTORC1-independent pathways in Δ*Lkb1* mice, as AMP kinase (AMPK), the target of Lkb1, regulates many signaling pathways besides mTORC1. In addition, there is no evidence to show that rapamycin restores bone phenotypes in Δ*Lkb1* mice. Unlike Lkb1, mTORC1 is a well-established target of TSC-TBC, and the phenotype of immature woven bone in Δ*Tsc1* mice was reversed by rapamycin, which further confirmed that bone changes in Δ*Tsc1* mice were the result of mTORC1 activation. Together, these findings demonstrate that differentiation of both preosteoblasts and osteoblasts is attenuated by mTORC1 activation.

Our data suggest that the impaired differentiation of preosteoblasts following mTORC1 activation is due to over-activated Notch signaling downstream. Notch signaling mediates communication between neighboring cells and decides their fate [32,33,34,35,53]. Specifically, gain of Notch function in preosteoblasts under the control of the type I collagen (*Col1a1*) promoter results in a phenotype similar to Δ*Tsc1* mice in the present study [32]. By inhibiting mTORC1 and the Notch pathway via rapamycin and DAPT/Si-*Notch1*, respectively, we showed that Notch acts downstream of mTORC1 and mediates the attenuation of osteoblastic differentiation by mTORC1. These findings are supported by a report which revealed that mTOR positively regulates Notch signaling in mouse and human cells through induction of the STAT3/p63/Jagged signaling cascade [28]. More recently, Wang et al. reported that mTORC1 activates Notch3 to accelerate the development of hypoxia-induced pulmonary hypertension [30] and Karbowniczek M *et al*. found that mTORC1 activates Notch in tuberous sclerosis complex and *Drosophila* external sensory organ development [54]. In contrast, Notch has also been shown to promote mTORC1 signaling by increasing Raptor protein expression in rat hepatoma cells and primary mouse hepatocytes [55]. We speculate that the interaction between mTORC1 and Notch may depend on cell type. Our data also confirmed that Runx2 is negatively regulated by mTORC1 and identified the Notch pathway as the responsible mechanism. Notch was identified as a major mediator of mTORC1 signaling in the impairment of preosteoblast differentiation in the present study.

In summary, this study clarified the potential role of mTORC1 signaling in the regulation of preosteoblast proliferation and differentiation and identified Notch signaling and Runx2 as critical downstream mediators. Pharmaceutical coordination of the pathways and agents in preosteoblasts may be beneficial in bone formation.

## Materials and Methods

### Cells

The preosteoblast cell line, MC3T3-E1, was maintained in alpha-MEM (Gibco) supplemented with 10% FBS (Gibco), 100 U/ml penicillin, and 100 mg/ml streptomycin sulfate, at 37°C with 5% CO2. For growth curve analysis, MC3T3-E1 cells were plated in six-well plates at a density of 8×10^4^ cells/well and cultured until confluent (8^th^ day). Growth rate was assessed by cell counting. Primary osteoblastic cells were prepared from the calvaria of 21-day-old fetal rats or newborn mice as described previously [56, 57, 58] and cultured using the same method as for MC3T3-E1 cells.

For osteogenic induction, 100 μg/ ml ascorbic acid (Sigma-Aldrich) and 10 mM β-glycerol phosphate (Sigma-Aldrich) were added to confluent cells. Rapamycin (Sigma-Aldrich) was added as stated in the Results section. Alizarin red staining was carried out according to standard techniques.

### Mice

The Southern Medical University Animal Care and Use Committee approved all procedures involving mice. Mice importing, transporting, housing and breeding were conducted according to the recommendations of "The use of non-human primates in research." Newborn C57BL/6 mice were purchased from the Laboratory Animal Centre of Southern Medical University. *Tsc1* ^flox/flox^ [59] and *Osx*-*GFP*::*Cre* [60] mice were both purchased from The Jackson Laboratory. The background of *Tsc1* ^flox/flox^ mice is 129S4/SvJae, and *Osx*-*GFP*::*Cre* mice were backcrossed on to a 129S4/SvJae background for 8 generations prior to use. We performed genotyping using genomic DNA isolated from tail biopsies, and the primers used are shown in S1 Table. The specificity of recombination was examined by PCR using primers flanking the floxed allele (S1 Table). Mice were sacrificed by cervical dislocation to ameliorate suffering.

### Preparation of decalcified sections, histochemistry and immunohistochemistry (IHC) and histomorphometric analyses

Femur tissues dissected from the mice were fixed using 4% paraformaldehyde in PBS at 4°C for 24 hours and then decalcified in 15% EDTA (pH 7.4) at 4°C for 14 days. The tissues were embedded in paraffin or optimal cutting temperature (OCT) compound (Sakura Finetek), and 2–5 μm sagittal-oriented sections were prepared for histological analyses. H&E and Toluidine blue staining was performed as previously described [61]. Tartrate-resistant acid phosphatase (TRAP) or alkaline phosphatase (ALP) staining was performed using a standard protocol (Sigma-Aldrich). For IHC, we incubated primary antibodies which recognized mouse phospho-S6 ribosomal protein (Ser235/236) (Cell Signaling, 1:100, #2211), proliferating cell nuclear antigen (PCNA) (Cell Signaling 1:200, #13110), osterix (Abcam, 1:500, ab22552), osteocalcin (Abcam, 1:500, ab93876), and Runx2 (Cell Signaling, 1:100) overnight at 4°C. All sections were observed and photographed on Olympus BX51 microscope. Immunohistochemical staining was evaluated by cell number counting and computerized optical density (OD) measurements. In proliferation analysis, osteoblast proliferation fraction was calculated as BrdU^+^ osteoblasts per total osteoblasts on bone surface. Osteoblasts on bone surface were discerned by morphology and calculated by two independent observers blinded to the groups. In immunohistochemistry assays, cells per bone perimeter (B.Pm) was used to calculate the number of positive cells, and integrated optical density per area of positive cells (IOD/area, mean density) was used to quantify the staining intensity by detecting in 6 different images taken at 100x magnification with Image Pro Plus 6.0 software (Media Cybernetics, MD,USA)[62]. Briefly, positively stained regions of the image were selected by HSI (hue, saturation and lightness) with S from 0–255, I from 0–210 and H from 0–25, and then the brown color was converted into grayscale signal. The grayscale signal was measured as mean optical intensity of staining (mean density) within the tissue masks. At least three mice per group were examined. Three equidistant sections spaced at 200 μm apart throughout the midsagittal section of femur were evaluated.

### X-ray and micro-CT analysis

The narcotized mice were analyzed using X-ray radiography. Quantitative analysis was performed in mice femora at 12 μm resolution on a micro-CT Scanner (Viva CT40; Scanco Medical AG, Bassersdorf, Switzerland) [63]. Briefly, scanning was performed at the lower growth plate in the femora and extended proximally for 300 slices. We started morphometric analysis with the first slice in which the femoral condyles were fully merged and extended for 100 slices proximally. Using a contouring tool, we segmented the trabecular bone from the cortical shell manually on key slices, and morphed the contours automatically to segment the trabecular bone on all slices. The three-dimensional structure and morphometry were constructed and analyzed for BV/TV (%), BMD (mg HA/mm^3^), Tb.N. (mm^–1^), Tb.Th. (mm) and Tb.Sp (mm). We also performed micro CT imaging in the mid-diaphysis of the femur and performed mid-shaft evaluation of 100 slices to quantify the cortical thickness, bone mineral density and outer/inner perimeter of the mid-shaft.

### Western blot

Cells and tissues were lysated by 2% sodium dodecyl sulfate with 2 M urea, 10% glycerol, 10 mM Tris-HCl (pH 6.8), 10 mM dithiothreitol and 1 mM phenylmethylsulfonyl fluoride. The lysates were centrifuged and the supernatants were separated by SDS-PAGE and blotted onto a nitrocellulose (NC) membrane (Bio-Rad Laboratories). The membrane was then analyzed using specific antibodies and visualized by enhanced chemiluminescence (ECL Kit, Amersham Biosciences).

### Preparation of undecalcified histology sections

To label the mineralization fronts, 10-week-old mice were subcutaneously injected with calcin (Sigma, 15 mg/kg body weight) in 2% sodium bicarbonate solution 10 days and 3 days before death [25]. After dissection, the femurs were fixed in 4% paraformaldehyde for 24 hours. They were then dehydrated through a graded series of ethanol (70–100%) and xylene before being embedded in methylmethacrylate (MMA) without prior decalcification [64]. 5 μm-thick sections were prepared for Goldner’s-Masson trichrome [65], and 10 μm-thick sections were prepared for double-labeling fluorescent analysis.

### Bromodeoxyuridine incorporation

After initial culture for 48 hours, primary calvarial cells were replated and expanded for an additional 24 hours. The cells were then treated with BrdU labeling reagent (Invitrogen) for 6 hours according to the manufacturer’s instructions and washed with PBS. The cells were fixed with 70% ethanol for 25 min at 4°C, and then stained for immunocytochemical analysis. Three to five areas for each group (n = 3 slides) were counted by two independent observers blinded to the groups. BrdU-positive cells were scored visually. For *in vivo* proliferation analysis, mice were injected with BrdU (1ml/100g body weight) 2 hours before sacrifice.

### Scanning electron microscopy (SEM)

The surface of the MMA embedded femurs were polished and acid-etched with 37% phosphoric acid for 2–10s. After washing for 5 min with 5% sodium hypochlorite they were coated with gold and palladium before examining with SEM (S-3700N, Hitachi, Japan).

### Doxycycline treatment

To prevent the *Osx* promoter from driving *Cre* expression, pregnant mice were exposed to 200 μg/ml doxycycline (Sigma-Aldrich) in drinking water until their progeny had been processed for calvarial cell isolation. Doxycycline 100 μg/ml was added sequentially to the isolated cells until they reached confluence.

### siRNA knockdown

We transiently transfected cells with siRNA using Lipofectamine RNAi MAX (Invitrogen, Carlsbad, CA, USA) in Opti-MEM medium (Invitrogen), according to the manufacturer’s instructions. The efficiency of transfection was measured by western blot. The sequences of siRNA used in this study were as follows: *Notch1*: sense: 5’-CCAAGAAGUUCCGGUUUGATT-3’, and anti-sense: 5’-UCAAACCGGAACUUCUUGGTT-3’; *STAT3*: 5’-CTGGATAACTTCATTAGCA-3’; *p63* (all isoforms): 5’-CACAGACCACGCACAGAAUdTdT-3’, 5’-UCCAGAUGACUUCCAUCAAdTdT-3’ (1:1 ratio mixture). Non-specific siRNA sequences were used as negative controls: sense: 5’-UUCUCCGAACGUGUCACGUTT-3’, and anti-sense: 5’-ACGUGACACGUUCGGAGAATT-3’. (GenePharma, Shanghai, China).

### *In vitro* kinase assay for mTORC1

Primary calvarial cells were lysed in ice-cold buffer (40mM HEPES (pH 7.4), 2mM EDTA, 10mM pyrophosphate, 10mM glycerophosphate, 0.3% CHAPS, and one tablet of EDTA-free protease inhibitors (Roche, Basel, Switzerland) per 25 ml). Supernatants were incubated with anti-mTOR antibody for 2h at 4°C, followed by addition of 30 μl of 50% slurry of protein G Sepharose beads for another 1h. Beads were then washed four times with lysis buffer and once kinase buffer (25mM HEPES (pH7.4), 50mM KCl, 10mM MgCl2, 250 μM ATP). 0.4μg of recombinant GST-tagged full-length STAT3 peptide (Creative BioMart, #1496H) was added to 30μl kinase buffer. Kinase assays were performed for 30 min at 30°C, and terminated by the addition of the 2×SDS sample buffer followed by boiling for 5 min.

### EMSA

A total of 2μg of nuclear protein extracted from calvarial cells was incubated with biotin-labeled STAT3 binding-site DNA probe in binding buffer (EMSA kit; Thermo scientific) for 30 minutes at room temperature. The probe used for the reaction contains the STAT3 binding site of the *ΔNp63* promoter with a sequence of 5'-GGATTCCTATTTCCCGTACATAATATGGAT-3'. After incubation, the samples were separated on a 6% polyacrylamide gel in Tris-borate ethylenediaminetetraacetic acid, transferred onto a nylon membrane, and fixed on the membrane by ultraviolet cross-linking. The biotin-labeled probe was detected with streptavidin-horseradish peroxidase (EMSA kit; Thermo scientific). A probe lacking nuclear extracts was used as a negative control. The specificity of the identified STAT3-DNA binding activity was confirmed by using a 200-fold excess of unlabeled probe containing a same sequence. For supershift analysis, 1 μg monoclonal anti-phosphorylated STAT3 (S727) (Cell Signaling Technology) was incubated with nuclear extracts for 30 minutes before the addition of the biotin-labeled DNA probe.

### Statistics

All results are presented as the mean ± S.D. Curve analysis was determined using Prism (GraphPad). The data in each group were analyzed using unpaired, two-tailed Student’s t-test. The level of significance was set at P < 0.05.

## Supporting Information

S1 Fig Alizarin red staining of rapamycin-treated fetal rat calvarial cells on the 14^th^ day of osteogenic induction. Image representative of three biological replicates is shown.
(TIF)

S2 Fig Rapamycin treatment reduced osteoclast number in C57BL/6 mice. (A) TRAP staining of distal femur from 10-week-old C57BL/6 mice treated with vehicle or rapamycin. (B) The number of osteoclasts (N.OC) on bone surface (/B.Pm) was measured. Scale bar, 100 μm. Data are presented as mean ± SD (n = 5 mice). ***P < 0.001 by t test.
(TIF)

S3 Fig Pale bones resulted by thickened cortical bone in Δ*Tsc1* mice. (TIF)

S4 Fig Δ*Tsc1* calvarial cells showed increased cellular proliferation. (A) BrdU staining of osteoblastic cells from P3 calvaria. (B) Percentage of BrdU positive cells out of total cells was measured. Data are presented as mean ± SD (n = 5).*P<0.05 by t test.
(TIF)

S5 Fig Scanning electron microscopy (SEM) analyses of distal femur bone surface. The osteoblasts of the Δ*Tsc1* mice showed abnormal shape and loss of osteoblast processes, appearing immature and poorly differentiated. Scale bar, 100 μm.
(TIF)

S6 Fig The number of osteoclast was reduced in Δ*Tsc1* mice. (A) TRAP staining of distal femur from 10-week-old Δ*Tsc1* and control mice. (B) The number of osteoclasts (N.OC) on the bone surface (/B.Pm) was measured. Data are presented as mean ± SD (n = 5). ***P<0.001by t test. Scale bar, 100 μm.
(TIF)

S7 Fig Differentiating MC3T3-E1 cells were treated with vehicle (V) or DAPT (D) and then subjected to immunoblotting for osteocalcin (A) on the 7^th^ and 14^th^ day and alizarin red staining (B) on the 14^th^ day. Differentiating control (C) and Δ*Tsc1* (Δ) primary calvarial cells were treated with vehicle (V) or DAPT (D) and then subjected to immunoblotting for osteocalcin (C) and alizarin red staining (D) on the 14^th^ day.
(TIF)

S8 Fig Model for effects of mTORC1 in proliferation and differentiation of preosteoblasts. mTORC1 accelerates proliferation of preosteoblasts by increasing expression of cyclin D1 and PCNA and inhibits differentiation and maturation of preosteoblasts by suppressing Runx2 due to activating of the Notch pathway.
(TIF)

S1 Table PCR primers. (DOCX)
