# Supplementary figures and images for: mTORC1 Prevents Preosteoblast Differentiation through the Notch Signaling Pathway
Source: PLoS Genet. 2015 Aug 4;11(8):e1005426. doi: 10.1371/journal.pgen.1005426 (PMC4524707; doi:10.1371/journal.pgen.1005426)

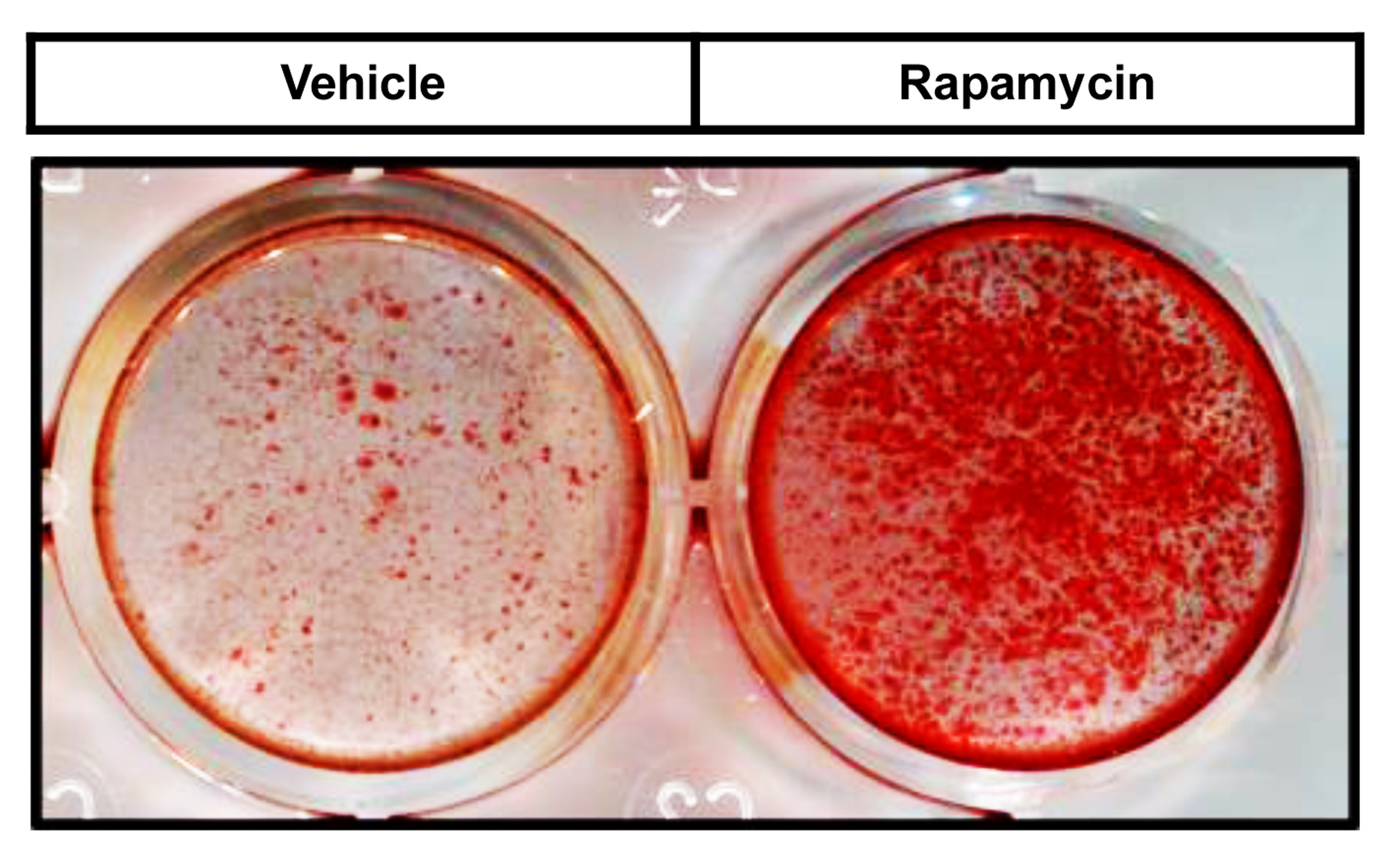

Supplement: S1 Fig — Image representative of three biological replicates is shown. (TIF) [file pgen.1005426.s001.tif]

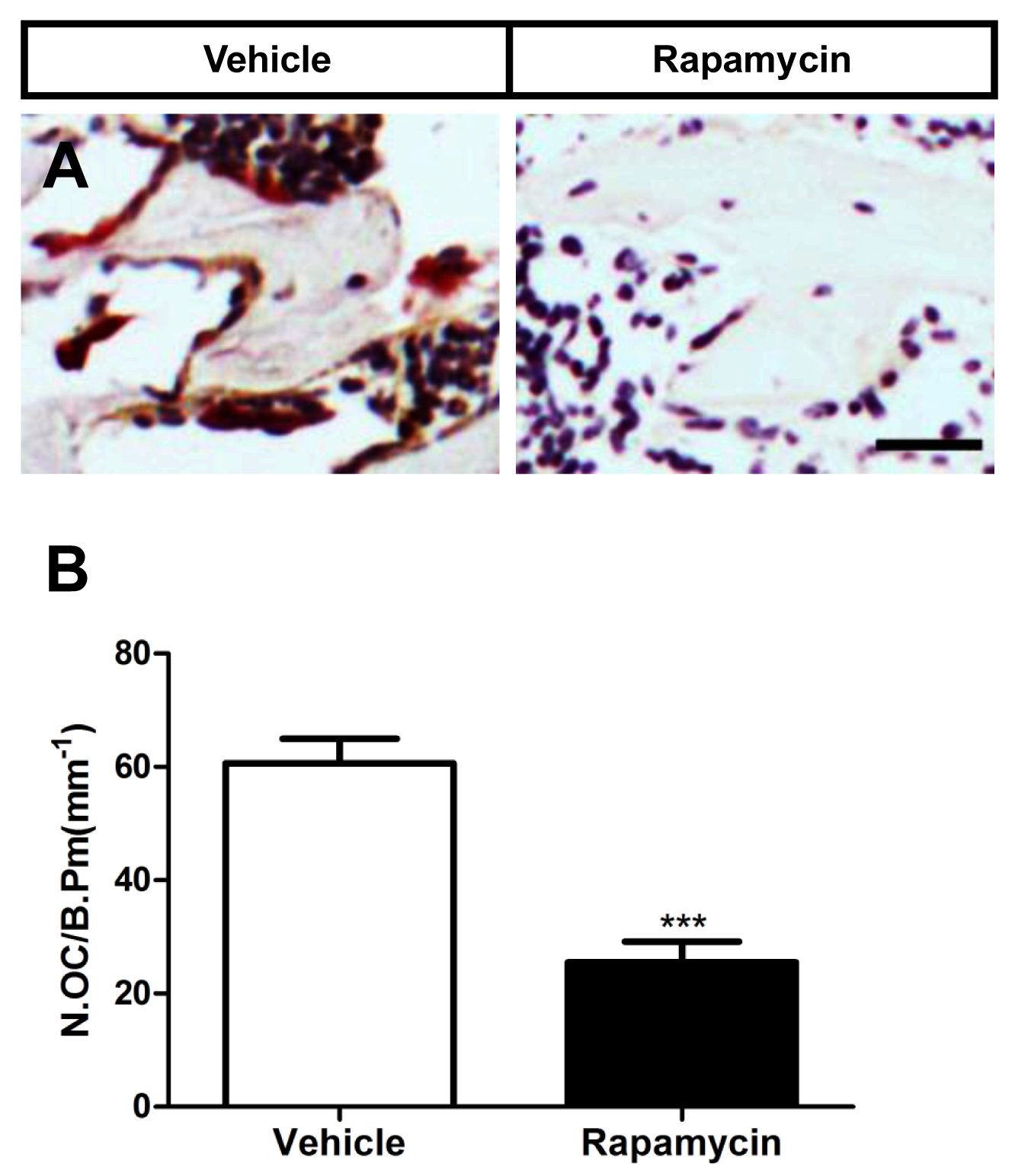

Supplement: S2 Fig — (A) TRAP staining of distal femur from 10-week-old C57BL/6 mice treated with vehicle or rapamycin. (B) The number of osteoclasts (N.OC) on bone surface (/B.Pm) was measured. Scale bar, 100 μm. Data are presented as mean ± SD (n = 5 mice). ***P < 0.001 by t test. (TIF) [file pgen.1005426.s002.tif]

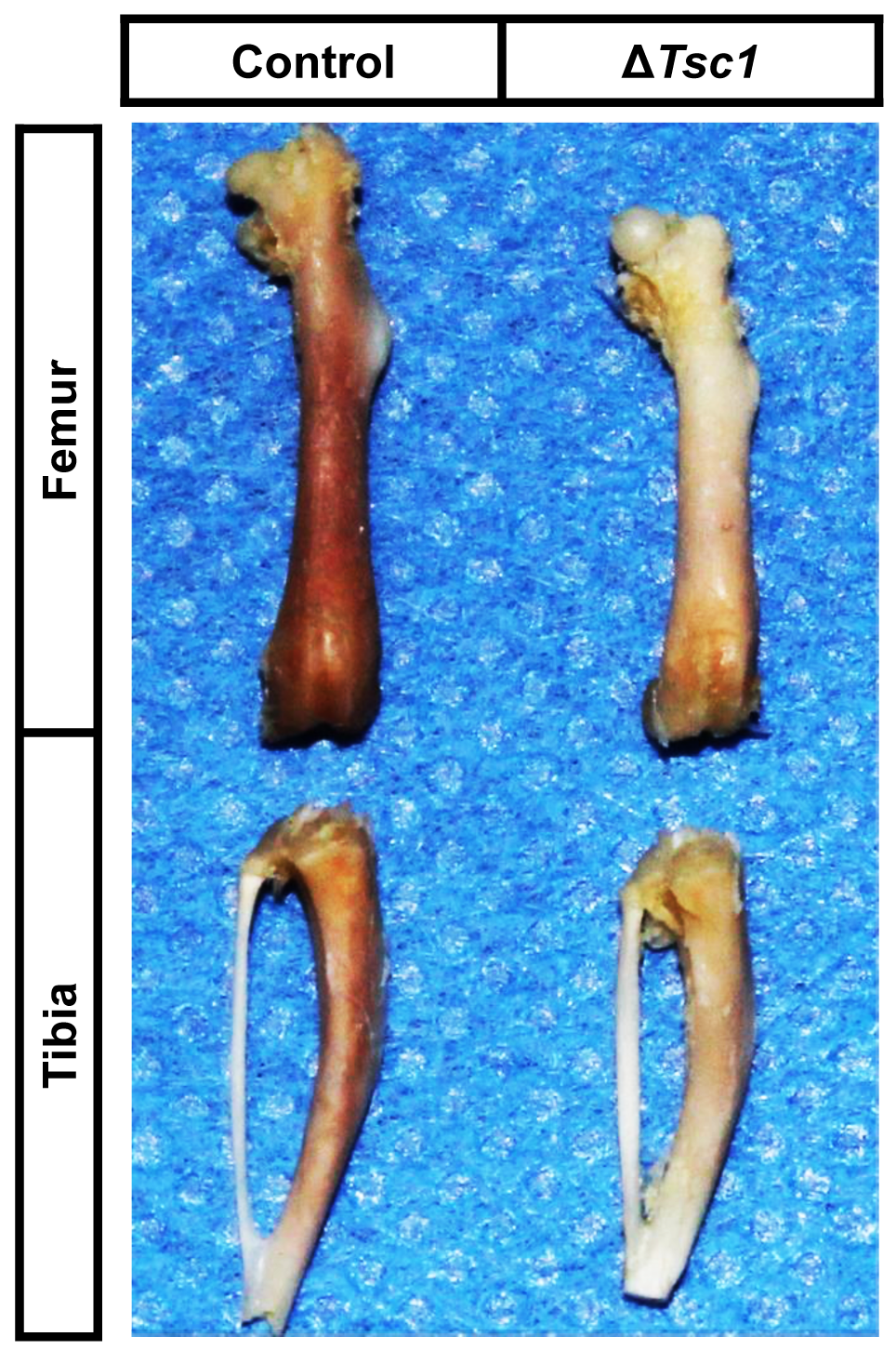

Supplement: S3 Fig — (TIF) [file pgen.1005426.s003.tif]

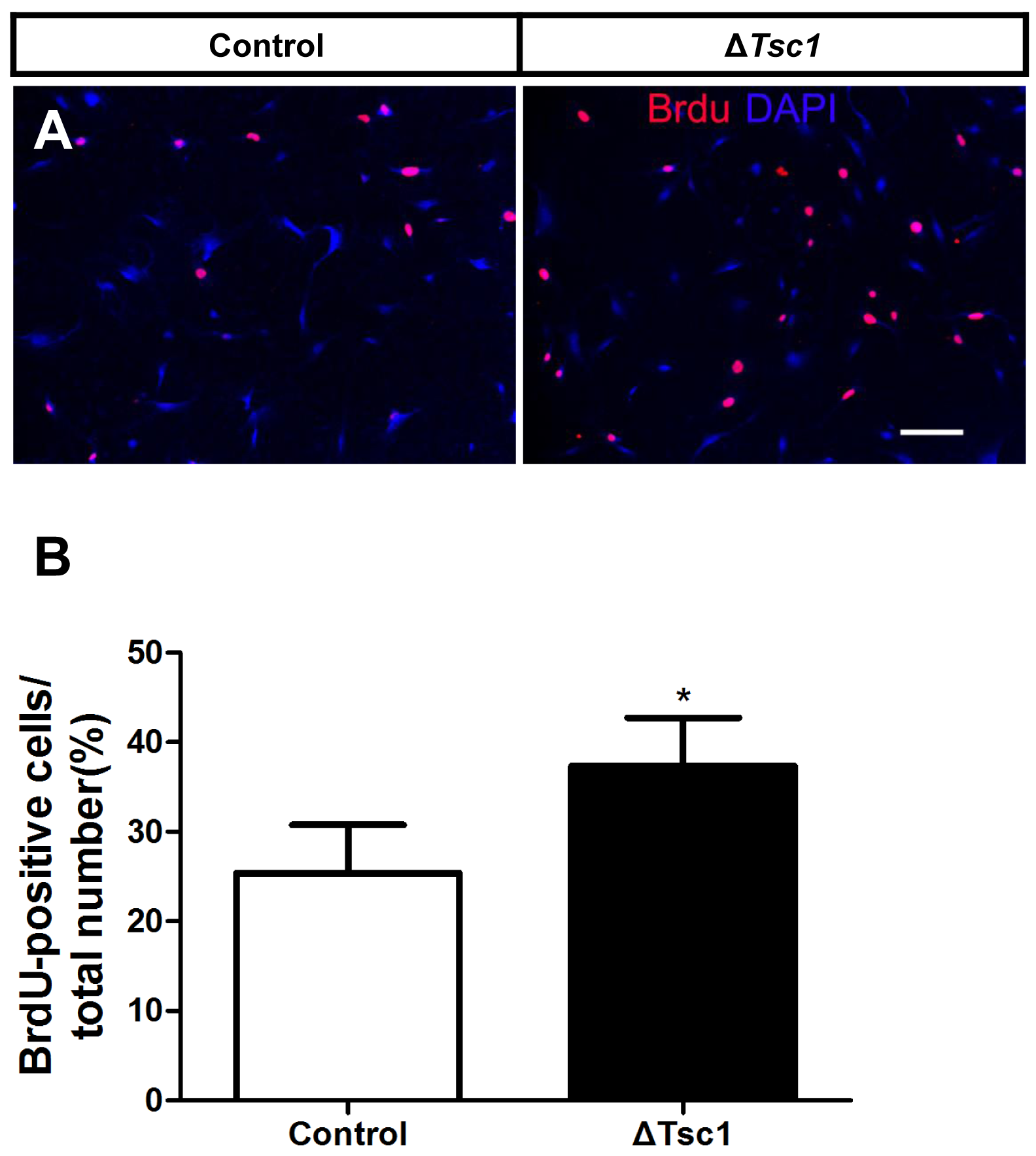

Supplement: S4 Fig — (A) BrdU staining of osteoblastic cells from P3 calvaria. (B) Percentage of BrdU positive cells out of total cells was measured. Data are presented as mean ± SD (n = 5).*P<0.05 by t test. (TIF) [file pgen.1005426.s004.tif]

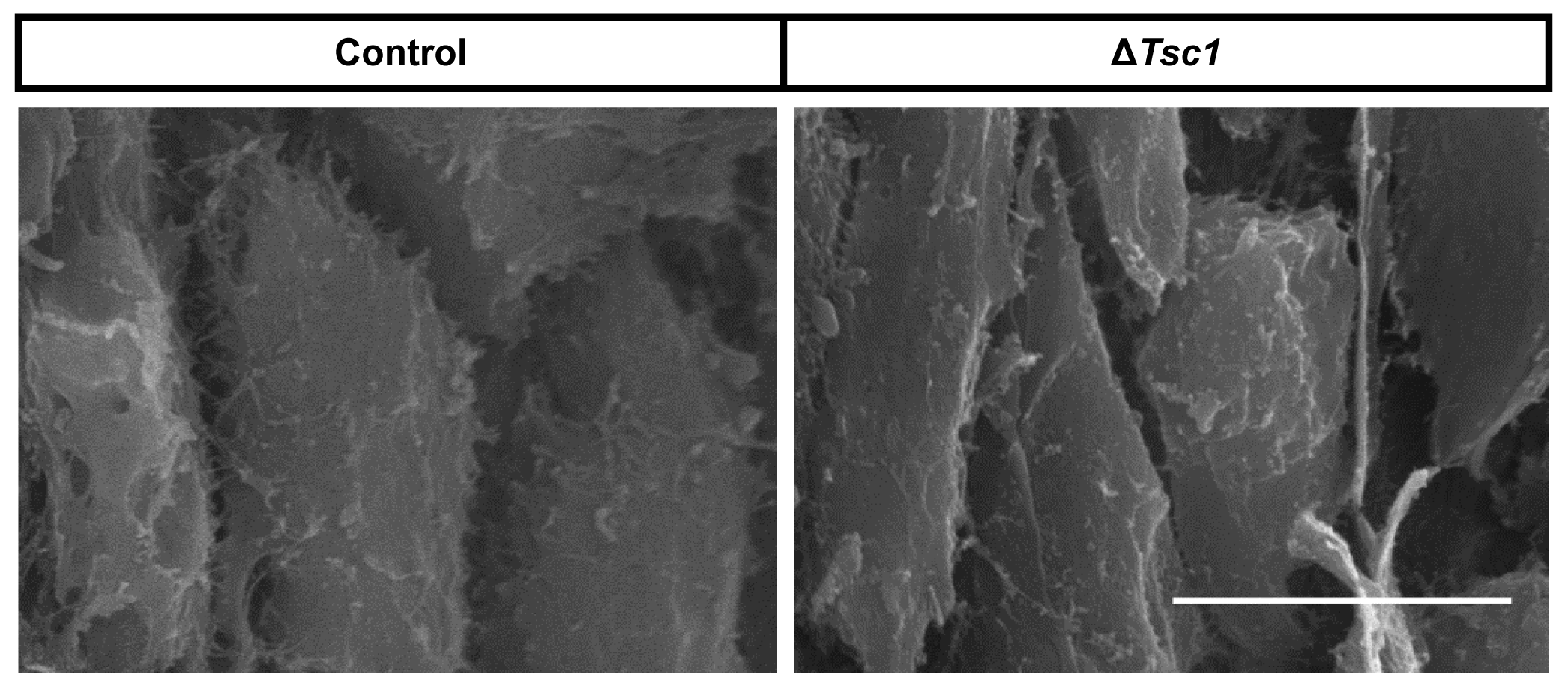

Supplement: S5 Fig — The osteoblasts of the ΔTsc1 mice showed abnormal shape and loss of osteoblast processes, appearing immature and poorly differentiated. Scale bar, 100 μm. (TIF) [file pgen.1005426.s005.tif]

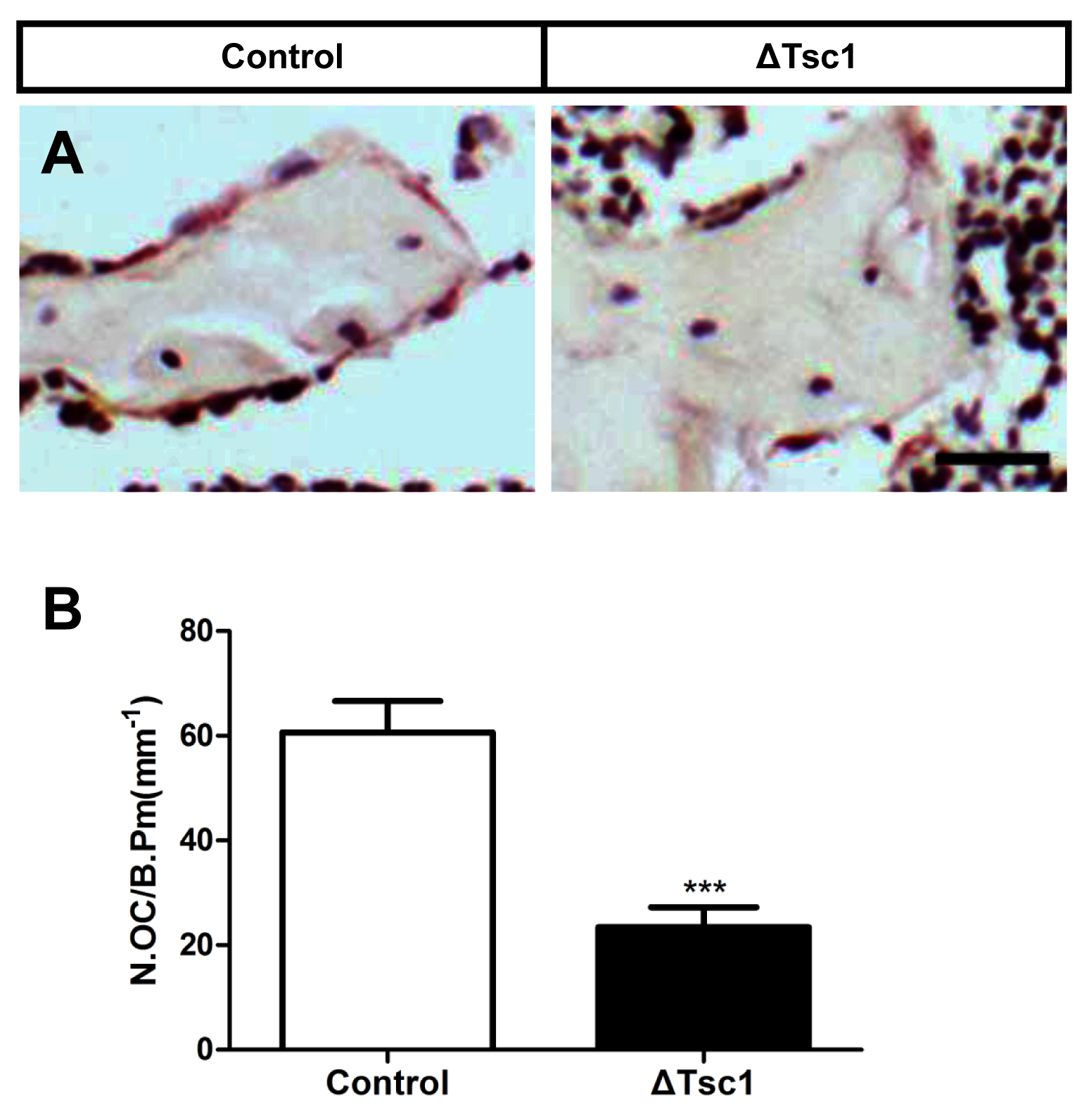

Supplement: S6 Fig — (A) TRAP staining of distal femur from 10-week-old ΔTsc1 and control mice. (B) The number of osteoclasts (N.OC) on the bone surface (/B.Pm) was measured. Data are presented as mean ± SD (n = 5). ***P<0.001by t test. Scale bar, 100 μm. (TIF) [file pgen.1005426.s006.tif]

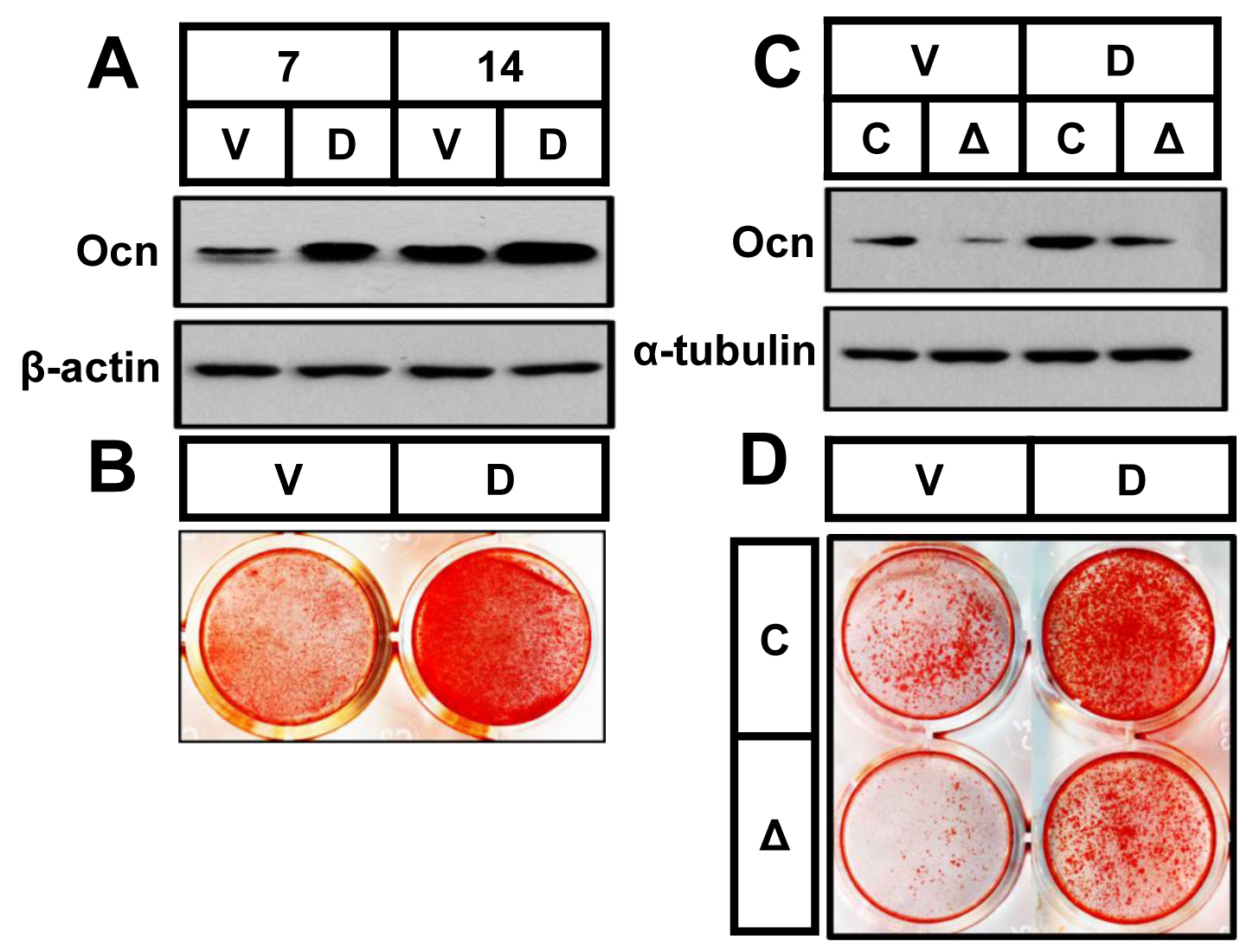

Supplement: S7 Fig — Differentiating control (C) and ΔTsc1 (Δ) primary calvarial cells were treated with vehicle (V) or DAPT (D) and then subjected to immunoblotting for osteocalcin (C) and alizarin red staining (D) on the 14th day. (TIF) [file pgen.1005426.s007.tif]

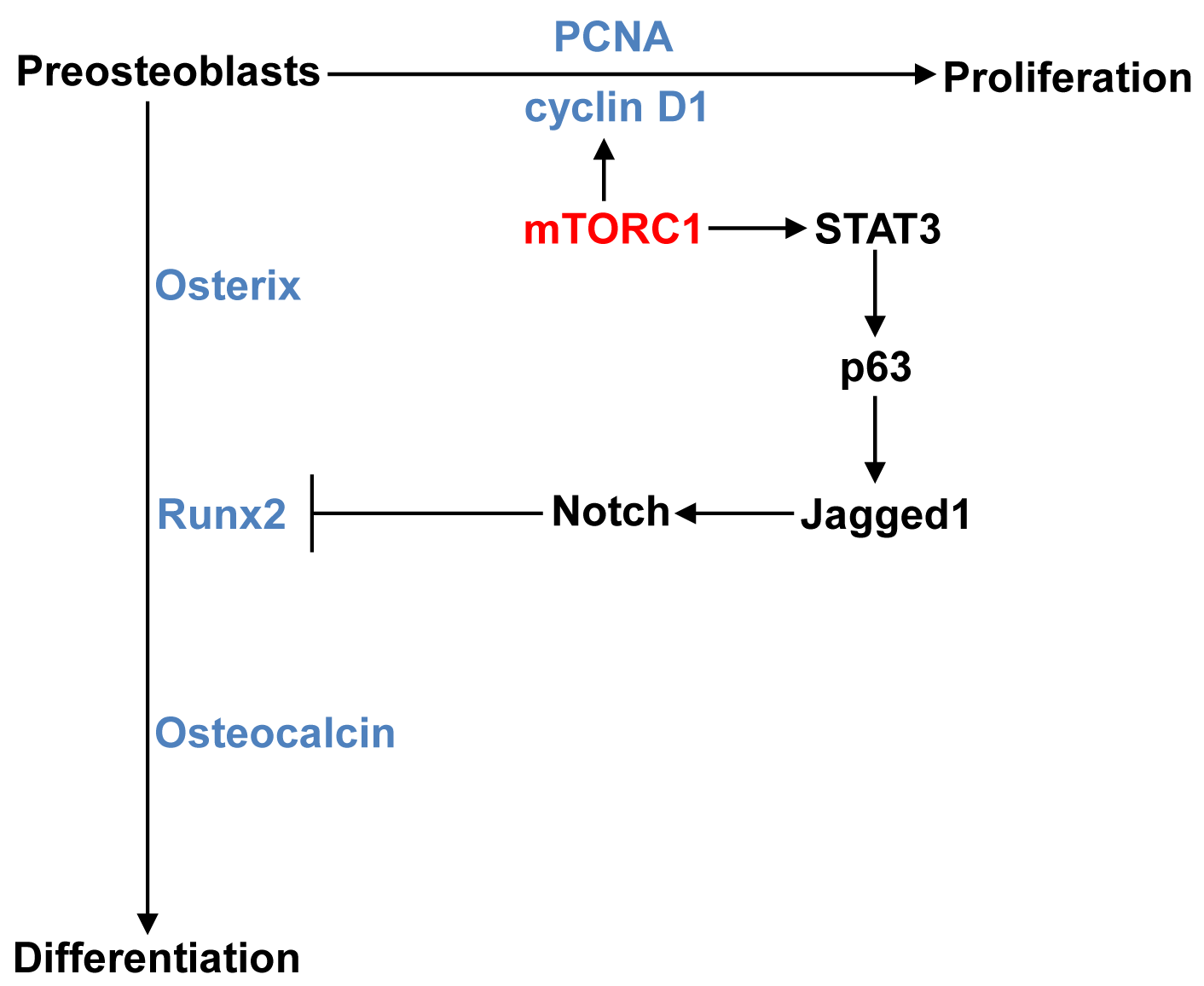

Supplement: S8 Fig — mTORC1 accelerates proliferation of preosteoblasts by increasing expression of cyclin D1 and PCNA and inhibits differentiation and maturation of preosteoblasts by suppressing Runx2 due to activating of the Notch pathway. (TIF) [file pgen.1005426.s008.tif]
