# Supplementary material for: mTORC1 Prevents Preosteoblast Differentiation through the Notch Signaling Pathway
Source: PLoS Genet. 2015 Aug 4;11(8):e1005426. doi: 10.1371/journal.pgen.1005426 (PMC4524707; doi:10.1371/journal.pgen.1005426)
Supplement: S1 Table — (DOCX) [file pgen.1005426.s009.docx]

**Table S1. PCR primers.**

| Gene | Strand | Sequence(5^，^to 3^，^) |
| --- | --- | --- |
| Generic *GFP* | Forward | AGT GCT TCA GCC GCT ACC |
|  | Reverse | GAA GAT GGT GCG CTC CTG |
| *Tsc1*^flox/flox^ | Forward | GTC ACG ACC GTA GGA GAA GC |
|  | Reverse | GAA TCA ACC CCA CAG AGC AT |
| Recombined *Tsc1* | F4536 | AGG AGG CCT CTT CTG CTA CC |
|  | R6548 | TGG GTC CTG ACC TAT CTC CTA |
| *GAPDH* | Forward | GCA CAG TCA AGG CCG AGA AT |
|  | Reverse | GCC TTC TCC ATG GTG GTG AA |
